# Associations of 2923 Olink proteins with demographic, lifestyle, environmental and health characteristics in middle-aged Chinese adults

**DOI:** 10.1007/s10654-025-01311-z

**Published:** 2025-10-10

**Authors:** Andri Iona, Baihan Wang, Jonathan Clarke, KaHung Chan, Maria G. Kakkoura, Charlotte Clarke, Neil Wright, Pang Yao, Mohsen Mazidi, Pek Kei Im, Maryam Rahmati, Christiana Kartsonaki, Sam Morris, Hannah Fry, Iona Y. Millwood, Robin G. Walters, Yiping Chen, Huaidong Du, Ling Yang, Daniel Avery, Dan Schmidt-Valle, Feifei Li, Canqing Yu, Dianjianyi Sun, Jun Lv, Michael Hill, Liming Li, Robert Clarke, Derrick A. Bennett, Zhengming Chen, China Kadoorie Biobank Collaborative Group

**Affiliations:** 1 Clinical Trial Service Unit, Nuffield Department of Population Health, University of Oxford, Oxford, UK; 2 NCDs Prevention and Control Department, Qingdao CDC, Qingdao, China; 3 Department of Epidemiology & Biostatistics, School of Public Health, Peking University, Beijing, China; 4 Peking University Center for Public Health and Epidemic Preparedness and Response, Beijing, China; 5 Key Laboratory of Epidemiology of Major Diseases (Peking University), Ministry of Education, Beijing, China; 6 Big Data Institute, University of Oxford, Old Road Campus, Oxford, OX3 7LF UK

**Keywords:** Exposure, Sex, Age, Frailty, Lifestyle, Proteomics, Biobank, Chinese

## Abstract

Previous studies have identified a large number of genetic variants affecting plasma levels of proteins, but little is known about the non-genetic factors influencing plasma levels of proteins in diverse populations. We measured plasma levels of 2923 proteins, using Olink Explore platform, in 2006 participants (mean age = 50.8 years; 62% female; mean body mass index = 23.9 kg/m^2^) in the China Kadoorie Biobank, without prior cardiovascular diseases. Linear regression analyses were used to assess the associations of individual proteins with 37 major exposures across multiple domains (e.g., socio-demographic, lifestyle, environmental, sample processing, reproductive factors, clinical measurements, and health-related indices), adjusted for potential confounders and multiple testing. These were further replicated and compared with findings in UK Biobank. Overall 31 exposures were associated with at least one protein, with age (*n* = 1154), sex (*n* = 827), body mass index (*n* = 869) showing the highest number of associations, followed by frailty index (*n* = 597), systolic blood pressure (*n* = 479), random plasma glucose (*n* = 387), ambient temperature (*n* = 292), and hepatitis B surface antigen positivity (*n* = 282), but with diet and physical activity showing little associations. Likewise, of the 2,923 proteins examined, 65% were associated with at least one exposure, with 25 proteins associated with ≥ 10 exposures, including five (CDH2, ADGRE2, ADGRD1, ACY1, MEGF9) after mutual adjustments. The patterns of associations were similar after further mutual adjustments for exposures examined but differed by sex, chiefly due to differences in lifestyle and reproductive factors. Most of the observed associations were replicated in the Europeans. For the purpose of Open Access, the author has applied a CC-BY public copyright license to any Author Accepted Manuscript version arising from this submission.

**Supplementary Information:**

The online version contains supplementary material available at 10.1007/s10654-025-01311-z.

## Introduction

Deciphering the human proteome could enhance our understanding of health and disease aetiology [1]. Plasma protein levels, secreted or leaked from cells or organs, may be affected by genetic and various non-genetic factors [2], and systematic investigation of plasma proteins-exposure relationships could improve our understanding of human biology and inform disease prevention and research strategies.

Traditionally, mass spectrometry has been used to measure plasma protein levels [3–5], but studies using this method are often limited by small sample sizes and low protein coverage [2]. In contrast, recent advances in affinity-based technologies (e.g., Olink and SomaScan) have enabled proteomics in large-scale population and clinical studies, allowing comprehensive investigations of relationships of plasma proteins with different health outcomes and associated traits [2, 6, 7]. In particular, the Olink platform, which utilises antibodies as reagents to bind target proteins, has been widely used in epidemiological research due to its high sample throughput, assay specificity and cost-effectiveness [6]. Recently, the Olink Explore 3072 platform was used to measure 2,923 plasma proteins in 54,219 participants in the UK Biobank [8], leading to many novel associations between proteins, demographic and clinical exposures and genetic factors [8]. It also replicated many of well-established associations for specific proteins, such as the associations between sex and LEP, and age and GDF15 [6–11]. Another affinity-based platform, SomaScan [7], has also been used in population-based and clinical studies, revealing proteomic associations with both genetic and non-genetic factors, such as age, sex, and adiposity [12–14].

Nevertheless, most previous proteomics studies investigated a relatively small number of proteins or only a few pre-selected exposures [15], without simultaneously considering a broader range of factors. Moreover, few have investigated associations of plasma proteins with composite indices (e.g., frailty) reflecting general lifestyle and health [16–19], which could be useful in population-level screening and disease prevention. Furthermore, previous studies typically focused on the discovery of protein quantitative trait loci (pQTLs) [8, 12, 13] for Mendelian Randomisation analyses and drug target discovery and validation [2], offering limited insights into the non-genetic factors influencing circulating proteins. Finally, evidence suggests protein concentrations vary across populations [20], driven not only by ancestry differences in the genetic architecture of the proteome, but also by differences in non-genetic factors, including environment. However, most large-scale proteomics studies have been conducted in European populations, highlighting the lack of diversity in proteomics research.

Exposome-wide association studies investigate the influence of a variety of life-course exposures from external and internal sources on phenotypic traits [21, 22]. When combined with proteomics, this method can offer important insights into the complex relationship between the exposome and proteome, as well as the biological mechanisms of how non-genetic factors impact human health [23]. Therefore, to fill the evidence gap, the present study aims to use the exposome-wide approach to [1] comprehensively explore the exposure profiles of ~ 3,000 Olink proteins in ~ 2,000 Chinese adults in the CKB; [2] assess the consistency of proteomic associations between the Chinese and European populations; and [3] identify priorities for future research.

## Methods

### Study population and design

The CKB is a prospective cohort study of > 512,000 adults who were recruited from 2004 to 2008 in 10 geographically diverse areas [24, 25]. At baseline, detailed information was collected from all participants using laptop-based questionnaires, including socio-demographic characteristics, medical history, and lifestyle habits, in addition to physical measurements including body composition and blood pressure. Non-fasting (with time since the last meal recorded) blood samples were also collected, processed, aliquoted, and then stored in liquid nitrogen for future unspecified research use.

Ethical approval were granted and maintained by the relevant institutional ethical research committees in the UK (Oxford Tropical Research Ethics Committee) and China (China CDC, Chinese Academy of Medical Sciences and Peking University). All participants provided written informed consent.

The present study utilised a case-subcohort design for IHD, including 1,951 cases and 2,026 subcohort participants with no prior cardiovascular disease to minimize biases arising from existing conditions’ effects on proteomic profiles [26]. The subcohort participants were randomly selected from 76,056 genotyped participants who were unrelated, passed quality control, and their plasma samples had not been previously reformatted. Random sampling was conducted using a reproducible pseudo-random number generation method (using the ‘sample’ function in R) [27].

### Proteomic assays

The plasma samples of all 3,977 participants collected at baseline were assayed using the Olink Explore 3072 platform, targeting 2,923 unique proteins. Samples were retrieved from liquid nitrogen, thawed, and aliquoted into 96-well plates (including 8 wells per plate for external QC samples) before being shipped to Olink laboratories in Uppsala, Sweden (1,472 proteins) and Boston, USA (1,469 proteins) for proteomic profiling. Protein levels were normalised by Olink to account for technical variations and provided in the arbitrary Normalized Protein eXpression (NPX) unit on a log2 scale. Six proteins were replicated across panels and showed high correlations (*r* > 0.8), so only one measure per protein was retained. Details on individual proteins are shown in **eTable 1**, with further assay information previously described [26, 28].

### Selected baseline characteristics

For the present analyses, a carefully selected set of 37 exposures was included to represent key domains relevant to the study objectives while avoiding collinearity of associated exposures within the same domain and minimizing redundancy. These 37 exposures were grouped into 6 broad categories (eTable 2), covering demographics (e.g., age, sex, study area), lifestyle habits (e.g., alcohol, smoking, diet, physical activity), environmental factors (e.g., outdoor temperature, fasting time), health and wellbeing (e.g., prior disease and mental health), clinical measures (e.g., BMI, SBP, RPG) and female reproductive factors (e.g., age at menarche, age at menopause, parity). We also computed a lifestyle index (ranging from 0 to 5, with a higher score indicating a healthier lifestyle) based on smoking, alcohol intake, physical activity, dietary habits, and body shape, which was previously developed in CKB and shown to be associated with multiple chronic diseases and life expectancy [17–19]. Similarly, a frailty index based on an accumulation of age-related deficits was computed considering medical conditions (based on self-reports of diagnosis by a doctor or physical measurements), symptoms, signs, and physical measurements, of which the procedure is described in a previous publication [16, 29].

### Statistical analyses

The main analyses were restricted to 2006 subcohort participants only (after excluding 20 participants with missing data on outdoor temperature). The prevalence or mean values of selected baseline variables were standardised to the age (5-year groups), sex, and study area. Plasma protein levels were standardized (i.e. values divided by their SD) and analysed as continuous variables. Linear regression was used to examine the associations of individual baseline characteristics with protein biomarkers, adjusting, in the main models, for age, quadratic term of age, sex, study area (10 areas), fasting time, quadratic term of fasting time, outdoor temperature, quadratic term of outdoor temperature, and plate ID. The quadratic terms were included to account for potential non-linear associations with protein levels. To further assess the independence of the observed associations with specific exposures, we also included the most relevant exposure variables (i.e., education, employment, income, ownership index, alcohol consumption, smoking, food diversity score, physical activity, BMI, SBP, self-rated health, diabetes, life satisfaction, mental disorder, exhale CO), in the mutually adjusted models (i.e., multivariable models including all aforementioned covariates in the same regression model), where appropriate. These variables were selected based on their relevance, lower collinearity, and availability across participants. For both the original (i.e., adjusted for age, quadratic term of age, sex, study area, fasting time, quadratic term of fasting time, outdoor temperature, quadratic term of outdoor temperature, and plate ID) and mutually adjusted models, when a particular variable or composite variable was considered as the exposure of interest, neither it nor the variables used to derive it were included in the model. Additionally, we performed sex-specific analyses to identify unique associations in females and males.

As many proteins and exposures are correlated, we followed the approach by Gadd et al. (2023) to correct for multiple testing [30]. Principal component analysis was conducted in the subcohort for 2,923 unique proteins and 32 exposures measured in both sexes, identifying 834 and 21 PC’s, respectively, that explained 90% of the cumulative variance (eFig. 2 and eTable 3). For overall and sex-specific analyses, an additional five largely independent reproductive exposures (available only in females) were included, resulting in a total of 26 exposures. Based on 834 protein PC’s and 26 exposures, a Bonferroni-adjusted p-value threshold was derived: (0.05/(834 × 26) = 2.305 × 10^− 6^) and applied across all linear regression models. The same Bonferroni-adjusted significance threshold was applied in the sex-specific analyses to ensure consistency and comparability of results across all models. Complete-case analysis (pairwise deletion) was used for the small number of variables with missing values (heating fuel [*n* = 919], cooking fuel [*n* = 478], rapeseed oil [*n* = 599], HBsAg+ [*n* = 21], RPG [*n* = 20]).

We also undertook separate analyses of the same 2923 Olink proteins (Olink Explore 3072 platform) in approximately 35,000 Europeans from the UK Biobank to replicate the main study CKB findings regarding key baseline characteristics (i.e., age, sex, BMI, SBP, RPG and prevalent diabetes), with the exclusion of participants with prior CVD or use of cholesterol-lowering medication [8]. Where appropriate, analyses were adjusted for age, quadratic term of age, sex, assessment centre, fasting time, quadratic term of fasting time, and plate ID. Associations were deemed replicated if they maintained a consistent direction of effect sizes to those in the CKB cohort and met the predefined significance threshold (*p* < 2.305 × 10^− 6^).

All statistical analyses were performed using R version 4.1.2 [31] and packages ‘tidyverse’, ‘stats’, ‘circlize’, and ‘ggplot2’.

## Results

Among the 2,006 participants, the mean baseline age was 50.8 (SD 10.5) years, 62% were female and the mean BMI was 23.9 (3.4) kg/m^2^ (Table 1). Overall, 15% of participants were regular alcohol drinkers (men: 37%; women 3%) and 25% (men: 63%; women: 2%) were current smokers. The prevalence of prior diseases was similar in males and females, with 8% of participants having respiratory disease, 2% having kidney/liver disease or tested sero-positive for HBsAg, and 6% having diabetes (self-reported or screen-detected). Similar patterns with baseline characteristics were observed in the full CKB cohort (eTable 4).

**Table 1:** Baseline characteristics of participants and their associations with Olink protein biomarkers

Characteristics	Mean (SD) or percentage, %^a^						No. of significant associations ^b^		
	Female (*n* = 1,247)	Male (*n* = 759)	All (*n* = 2,006)				Female	Male	All
Demographics									
Age, years	50.7 (10.2)	50.8 (11.0)	50.8 (10.5)				1133	612	1154
Sex	─	─	─				─	─	827
Urban residents	52.0	48.6	50.6				224	185	359
Schooling (> 9 years)	20.4	7.6	22.9				4	0	3
Employed	60.1	77.5	66.8				1	0	3
Household income (≥¥20,000)	43.0	47.4	44.5				1	11	9
Ownership index ^c^	3.3 (1.3)	3.4 (1.4)	3.3 (1.3)				0	29	15
Lifestyle									
Regular alcohol drinker	2.6	37.2	15.3				0	53	75
Current smoker	2.3	63.3	25.3				5	47	53
Diet									
Food diversity score ^d^	11.4 (3.3)	11.3 (3.2)	11.3 (3.3)				0	0	1
Rapeseed oil	33.3	38.8	35.4				6	0	22
Physical activity, MET-hrs/day	20.5 (13.2)	23.2 (16.3)	21.4 (14.5)				1	0 0	3
Environmental									
Outdoor temperature, °C	16.0 (10.6)	15.7 (10.9)	15.9 (10.7)				174	57	292
Clean heating fuel	45.3	44.3	45.0				0	0	1
Clean cooking fuel	49.9	36.0	44.8				1	0	0
Health and wellbeing									
Self-rated health	8.5	8.2	8.3				0	0	3
Respiratory disease	8.3	8.0	8.2				0	2	0
Kidney/liver disease	2.1	2.5	2.2				0	2	0
HBsAg+	2.2	2.5	2.3				198	45	282
Diabetes	7.0	5.8	6.5				217	39	340
Cancer	0.6	0.6	0.7				2	1	1
Life satisfaction	3.7	4.9	4.0		3.7		1	0	0
Mental disorder	1.1	1.5	1.2		1.1		0	0	0
Clinical measurements									
BMI, kg/m^2^	24.0 (3.5)	23.7 (3.3)	23.9 (3.4)				576	353	869
Standing height, cm	154.5 (6.1)	165.8 (6.5)	158.7 (8.3)				6	0	18
SBP, mmHg	129.4 (22.2)	132.6 (19.9)	130.5 (21.4)				295	80	479
DBP, mmHg	77.2 (10.6)	79.7 (11.6)	78.0 (11.1)				178	108	380
Heart rate, bpm	79.4 (11.4)	78.0 (11.9)	78.8 (11.6)				57	41	234
Exhaled CO, ppm	5.0 (2.2)	11.7 (2.5)	7.5 (2.3)				0	35	29
FEV1/FVC ratio	85.1 ( 6.1)	84.9 (10.1)	85.0 (8.5)				0	1	1
RPG, mmol/L	6.1 (8.2)	5.9 (8.8)	6.0 (8.5)				254	84	387
Fasting time, hours	5.2 (5.0)	5.0 (5.0)	5.1 (5.0)				56	26	79
Reproductive factors									
Age at menarche, years	15.4 (2.0)	─	15.4 (2.0)				1	─	1
Age at menopause, years	39.2 (4.3)	─	39.2 (4.3)				2	─	2
Post-menopausal	54.7	─	54.7				181	─	181
Parity	99.8	─	99.8				4	─	4
Age at first live birth, years	23.9 (3.3)	─	23.9 (3.3)				0	─	0
Lifestyle index ^e^	3.1 (0.8)	2.2 (1.0)	2.8 (1.0)				99	126	342
Frailty index ^f^	0.1 (0.06)	0.1 (0.06)	0.1 (0.06)				279	102	597

^a^ Baseline characteristics adjusted for age (10-year age groups) and study area (10 regions).

^b^ Analyses are adjusted for age, age^2^, sex, study area, fasting time, fasting time^2^, outdoor temperature, outdoor temperature^2^ and plate ID, where appropriate. Bonferroni (PCA) corrected p-value < 0.05

^c^ 6-point index of qualitative measures of living standards

^d^ 24-point index of frequency of intake in 12 food groups

^e^ 5-point index of low-risk lifestyle characteristics

^f^Index derived from 28 variables of accumulation of health deficits and physical activity, on a scale from 0 to 1

*BMI* Body mass index; *CO* carbon-monoxide; SBP systolic blood pressure; *DBP* Diastolic blood pressure; *FEV1/FVC* Forced Expiratory Volume in 1 s / Forced Vital Capacity; *HBsAg+* Hepatitis B virus surface antigen seropositive; MET: Metabolic Equivalent of Task; *RPG* random plasma glucose

**Fig. 1:**
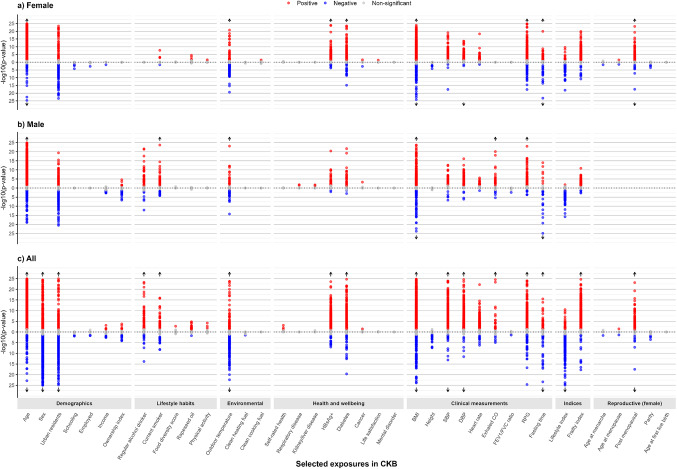
Exposure profiles with 2923 protein biomarkers in CKB, overall and by sex. Three Miami plots are presented: one for female-specific analysis, one for male-specific analysis, and one for overall analysis. The x-axis represents baseline characteristics grouped by category, while the y-axis shows the negative logarithm of the p-value (-log10 p-value) for the association between each exposure and protein biomarkers. Each dot represents the -log10 Bonferroni corrected p-value for these associations. For visualization purposes, -log10 p-values exceeding 25 are not displayed (indicated with arrow). Positive associations are shown in red, negative associations in blue, and non-significant associations in grey. Analyses are adjusted for age, age^2^, sex, study area, fasting time, fasting time^2^, outdoor temperature, outdoor temperature^2^ and plate ID, where appropriate. *BMI* Body mass index; *CKB* China Kadoorie Biobank; *CO* carbon-monoxide; SBP systolic blood pressure; *DBP* Diastolic blood pressure; *FEV1/FVC* Forced Expiratory Volume in 1 s / Forced Vital Capacity; *HBsAg+* Hepatitis B virus surface antigen seropositive; *RPG* random plasma glucose

**Fig. 2:**
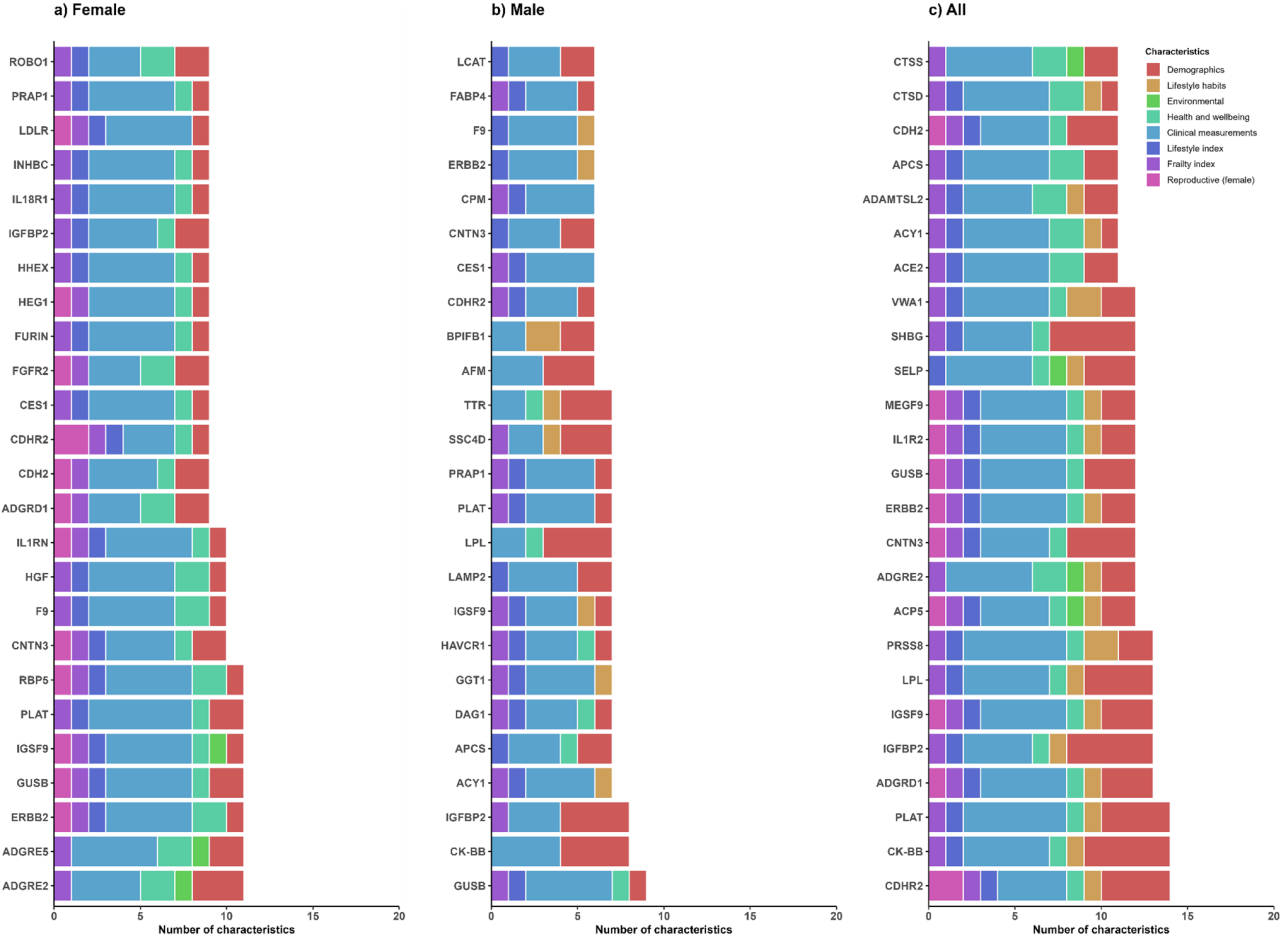
Exposure profiles by characteristics type of the top 25 protein biomarkers with most associations, overall and by sex. The bar plots show the number of baseline associated with the 25 most frequently associated protein biomarkers based on Bonferroni-corrected p-values. The analyses are presented separately for females, males, and overall. The x-axis represents the protein biomarkers, while the y-axis indicates the number of baseline characteristics associated with each protein. Bars are color-coded to represent different baseline characteristic groups. Analyses are adjusted for age, age^2^, sex, study area, fasting time, fasting time^2^, outdoor temperature, outdoor temperature^2^ and plate ID, where appropriate

Among 37 baseline characteristics examined, 31 were associated with at least one protein at the Bonferroni-adjusted threshold in the main model (Table 1; Fig. 1 and eTable 5). The four baseline characteristics that showed the largest number of associations with proteins were age (*n* = 1154), sex (*n* = 827) and BMI (*n* = 869), and frailty index (*n* = 597). Likewise, of the 2,923 proteins examined, 1900 (65%) were associated with at least one exposure, with three proteins (CDHR2, CK-BB, and PLAT) showing the most associations with baseline characteristics (*n* = 14), primarily involving demographic factors and clinical measurements (Fig. 2, eFig. 3). In the mutually adjusted model, the patterns of associations were similar, although there was a reduced number of significant associations for age (*n* = 766), sex (*n* = 467), BMI (*n* = 675), frailty index (*n* = 573) and other exposures (eTables 5, 6, 7 and 8). After mutual adjustments, five proteins (CDH2, ADGRE2, ADGRD1, ACY1, MEGF9) remained to be associated with > 10 exposures.

**Fig. 3:**
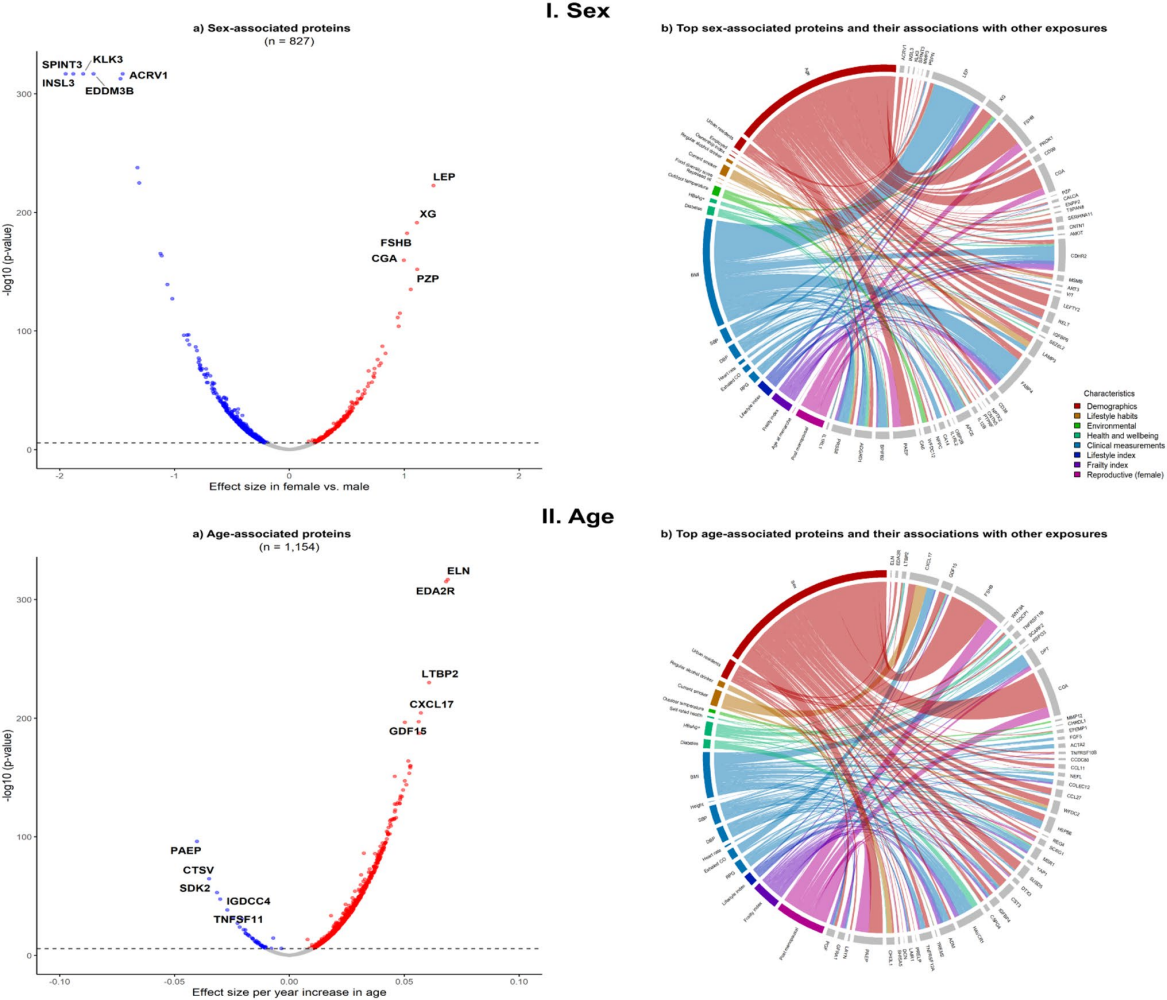
Sex- and age-associated protein biomarkers and their associations with other exposures. **a** and **c** represent the associations of sex and age, respectively, with protein biomarkers. The x-axis represents the effect size of the association between sex or age and the protein biomarkers, while the y-axis indicates the –log10 p-value. Red dots denote positive Bonferroni corrected associations, blue dots denote negative Bonferroni corrected associations, and grey dots denote non-significant associations. **b** and **d** illustrate the top sex- and age-associated protein biomarkers, respectively, and their associations with other exposures. The width of the ribbons is inversely proportional to the p-value, indicating the strength of the association (smaller p-values correspond to wider ribbons). The colors of the ribbons represent different baseline characteristic groups. The top protein biomarkers that are not associated with other exposures are not presented in the figure Analyses are adjusted for age, age^2^, sex, study area, fasting time, fasting time^2^, outdoor temperature, outdoor temperature^2^ and plate ID, where appropriate. *BMI* Body mass index; *CO* carbon-monoxide; SBP systolic blood pressure; *DBP* Diastolic blood pressure; *HBsAg+* Hepatitis B virus surface antigen seropositive; *RPG* random plasma glucose

Of the 827 sex-related proteins (higher levels in females for 259 and in males for 568 proteins), the strongest associations were with LEP, XG, FSHB, CGA, and PZP in females, and with ACRV1, EDDM3B, INSL3, SPINT3, and KLK3 in males (Fig. 3I.a). Among the top 50 sex-related proteins, most were also associated with other exposures, chiefly age (e.g., FSHB, CGA, XG, LEFTY2, RELT) and BMI (e.g., LEP, FABP4, CDHR2, APCS, CA14; Fig. 3I.b). Additionally, 77 sex-related proteins were not associated with other exposures examined, of which 27 were uniquely associated with female sex (e.g., CSF3, MELTF, ITIH4, CTRC, FCGR3B) and 50 with male sex (e.g., EDDM3B, TEX101, CRISP2, HS6ST2, SPESP1; eTable 8). Among women, some proteins were also associated with several reproductive factors (e.g. menopause status), the number of which changed little in mutually adjusted models (eTable 6).

Of the 1,154 age-related proteins, the strongest positive associations were with EDA2R, ELN, LTBP2, CSCL17, and GDF15, while the strongest negative associations were with PAEP, CTSV, SDK2, IGDCC4, and TNFSF11 (Fig. 3 II.a). Among the top 50 age-related proteins, most were also associated with other exposures, chiefly sex (e.g., FSHB, CGA, PAEP, CST3, SUSD5) and BMI (e.g., DPT, HSPB6, FGF5, MSR1, HAVCR1; Fig. 3 II.b). Additionally, 168 age-related proteins (e.g., ITGB5, IL17D, TIMP4, SORCS2, TNFSF13) were not associated with other exposures (eTable 8). In sex-specific analyses, age was associated with 1,133 proteins in males and 612 in females (Table 1; eFig. 4 I.a & II.b). Of the 524 overlapping proteins in both sexes, nearly all (> 95%) associations were directionally consistent, but stronger in females (*r* = 0.62; eFig. 5). Among females, FSHB, ELN, EDA2R, CXCL17, and LTBP2 showed the strongest positive associations with age, while PAEP, SDK2, CTSV, IGDCC4 and STC2 showed the strongest negative associations (eFig. 4 I.a). Among males, EDA2R, ELN, LTBP2, KLK4, and GDF15 showed the strongest positive associations, and EGFR, INSL3, IGFBP3, ERBB3, and TNFSF11 showed the strongest negative associations (eFig. 4 II.a). Among females the top age-related proteins were predominantly associated with menopause, while among males they were mainly associated with clinical measurements (i.e., BMI, SBP, and RPG), exhaled CO and current smoking (eFig. 4 I.b & 4 II.b). Additionally, 394 and 352 age-related proteins in females and males, respectively, were not associated with other exposures (eTable 8). In overall analyses, regular alcohol consumption and current smoking were associated with 75 and 53 proteins (4 overlapping), respectively, with MAMDC4, CHI3L1, VWA1, VCAN, and FGF21 most strongly associated with alcohol drinking and CXCL17, LAMP3, ALPP, MSLN and SCGB3A1 with smoking (Table 1, eFigure 6). Among men, most of these protein associations with alcohol (*n* = 53) and smoking (*n* = 47) were significant (eFigure 7). Overall, outdoor temperature was associated with 292 proteins (e.g., SNED1, SPINK6, LRP1, STX7, WFDC12), reducing to 174 in female- and 57 in male-specific analyses (eFigs. 6 and 7).

Among clinical measurements, BMI was associated with the largest number of proteins (869) followed by SBP (*n* = 479) and RPG (*n* = 387; 274 overlapping with prevalent diabetes; Table 1). In mutually adjusted models, the number of significant associations was reduced (eTable 5, eTable 6), especially for SBP (*n* = 157) and RPG (*n* = 54). For BMI, the strongest associations were with LEP, FABPA, IGFBP2, CK-BB, and GHR (eFigure 8a). In sex-specific analyses, BMI was associated with 576 proteins in females and with 353 in males, with similar strength of associations among 300 overlapping proteins (*r* = 0.97; Table 1 and eFig. 4b). The leading BMI-related proteins demonstrated similar profiles, with > 90% of them also associated with age, sex, prevalent diabetes, and other clinical measurements in overall and sex-specific analyses (eFigs. 8b, 9 I.b & II.b).

Overall, prevalent diabetes and HBsAg positivity were associated with 340 and 282 proteins, respectively (Table 1), with more associations in females than males (217 vs. 39 and 198 vs. 45, respectively; Table 1 and eFig. 7 h). These were reduced to 212 proteins for prevalent diabetes but increased to 292 for HbsAg positivity in the mutually adjusted models (eTables 5, 6). Other health-related measures, including self-rated health and prior cancer, were associated with fewer than three proteins. For females, post-menopause was associated with 181 proteins, with the strongest positive associations being with FSHB, CGA, DPP4, CHAD, and COL1A1 and the strongest inverse associations with PAEP, CHRDL2, SDK2 and C1QA, and RSPO1 (eFig. 10a). Additionally, the top menopause-related proteins were predominantly associated with age (eFig. 10b).

**Fig. 4:**
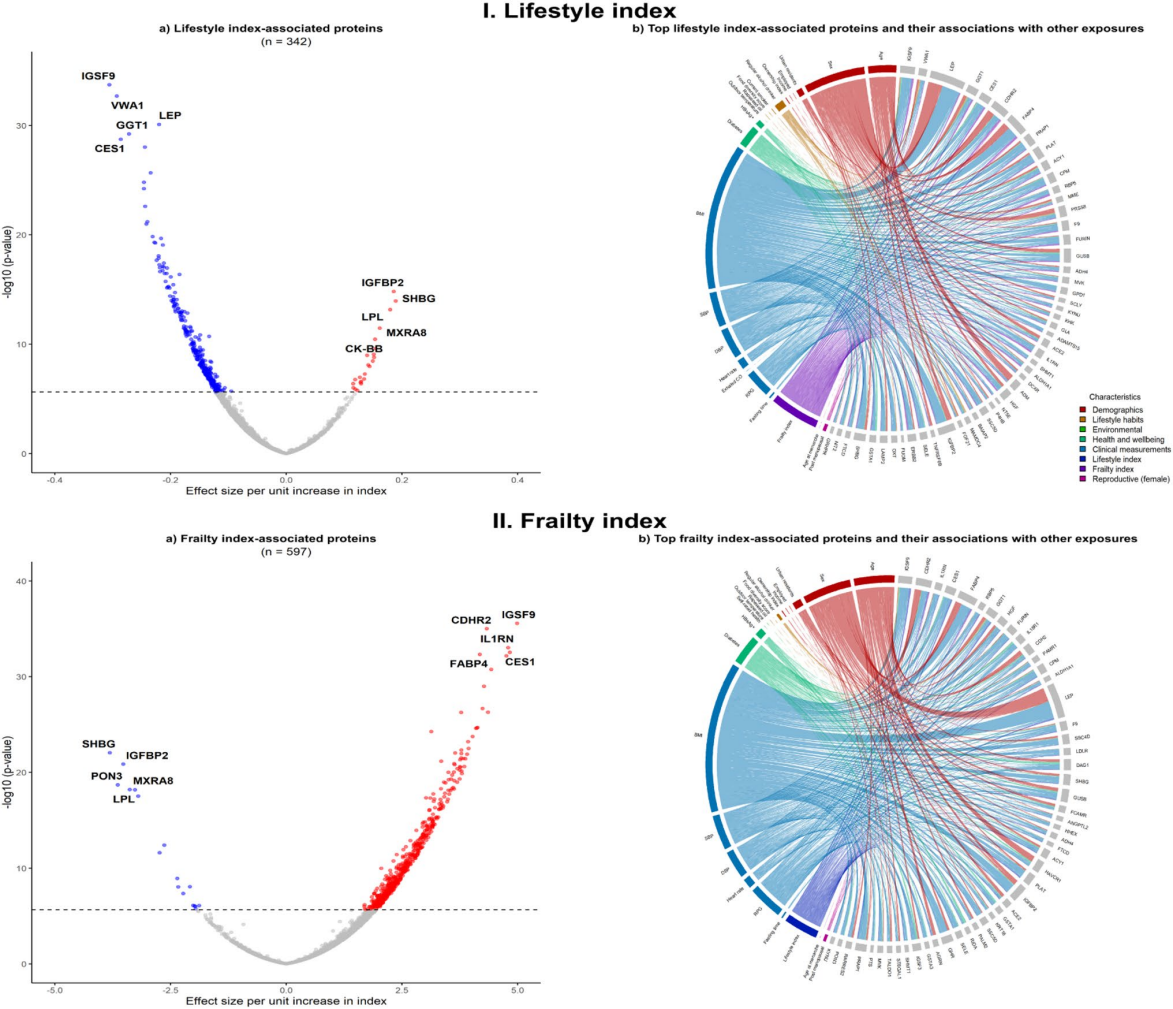
Lifestyle and frailty indices-associated protein biomarkers and their associations with other exposures. Symbols and conventions as in Fig. 3

Lifestyle index was associated with 342 proteins (e.g., IGSF9, VWA1, LEP, GGT1, CES1) in overall analyses, and with 126 and 99 proteins in male- and female-specific analyses (Table 1; Fig. 4I.a, eFig. 11 I.a & II.a), which was reduced to 188 proteins overall in the mutually adjusted model. Most of these proteins were also associated with the individual components of the index, particularly age, sex and clinical measurements, with alcohol and smoking being particularly notable among males (Fig. 4I.b, eFig. 11 I.b & II.b).

Overall, the frailty index was associated with 597 proteins (e.g., IGSF9, CDHR2, IL1RN, CES1, FABP4), with 300 (50%) proteins overlapping with the lifestyle index, albeit in opposite directions (*r*=-0.91; Table 1; Fig. 4c II.a). In the mutually adjusted model, there was only a small reduction in the number of significant proteins (*n* = 573). In sex-specific analyses, 102 and 279 proteins were associated with frailty index in females and males, respectively (eFig. 13 I.a & II.a). Many of the frailty index-related proteins were also associated with individual components of the index, including age, sex, clinical measurements, and prior diseases, with similar patterns in men and women (Fig. 4 II.b, eFig. 12 I.b & 12 II.b).

**Fig. 5:**
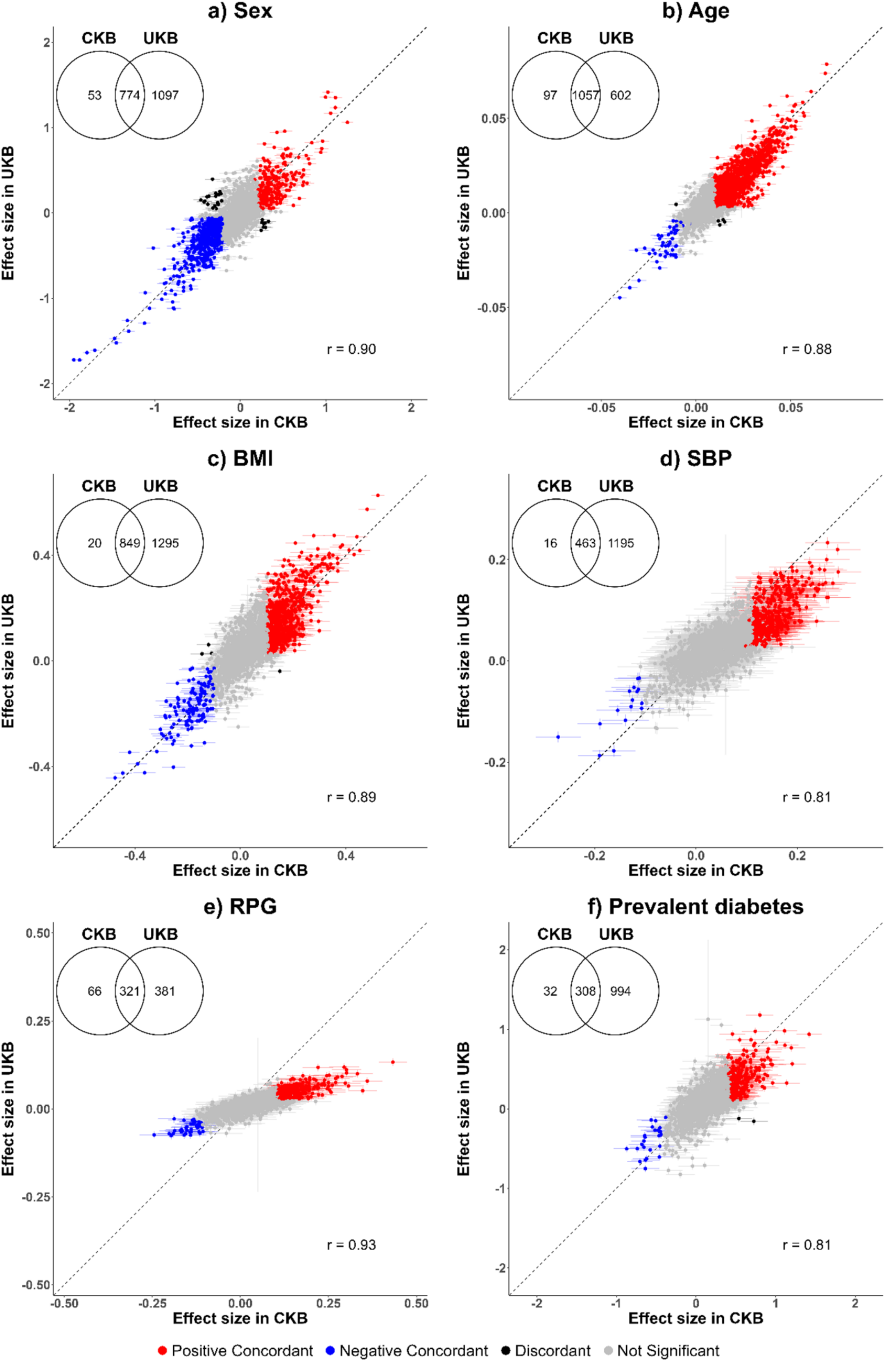
Associations of six selected key baseline characteristics with protein biomarkers in CKB and UKB. In CKB, analyses were adjusted for age, age^2^, sex, study area, fasting time, fasting time^2^, outdoor temperature, outdoor temperature^2^ and plate ID, where appropriate. In UKB, analyses were adjusted for age, age^2^, sex, assessment centre, fasting time, fasting time^2^, season, and plate ID, where appropriate. *BMI* Body mass index; *CKB* China Kadoorie Biobank; *SBP* systolic blood pressure; *UKB* UK Biobank; *RPG* Random plasma glucose

In UKB participants (mean age 57 [SD 8.1], 55% female), 1,871 proteins were significantly associated with sex, 1,659 with age, 2,144 with BMI, 1,658 with SBP, 702 with RPG, and 1,302 with prevalent diabetes (Fig. 5). Over 90% of the significant associations in CKB were replicated in UKB, except of RPG, which had a lower (~ 80%) replication rate. However, > 95% of RPG associations in CKB were replicated in UKB using HbA1c (eFig. 13). Moreover, overlapping significant proteins in CKB and UKB showed high correlations (*r* > 0.80) in effect sizes, with > 95% being directionally consistent. In sex-specific analyses, there were similar patterns and replication rates between the two populations (eFig. 14).

## Discussion

In this exposome-wide analysis of Olink proteins in Chinese adults, we identified a large number of associations between various exposures and levels of ~ 3,000 proteins. In particular, age, sex, BMI and frailty index each showed significant and apparently independent associations with plasma levels of 450–750 proteins. A range of other exposures including: socio-demographic, environmental factors, clinical measurements, health-related traits, and composite lifestyle and health indices, were also associated with levels of modest numbers of proteins. Many proteins were associated with multiple exposures, including 25 that showed associations with > 10 exposures. We also observed differences in proteomic-exposure associations between females and males, and replicated > 90% of proteomic associations with key exposures in the European populations.

A large number of proteins in our study showed associations with several different exposures, including some that remained associated with > 10 exposures even after adjustments for relevant covariates. These proteins may reflect the general and pervasive impact of the exposome on human health, as reflected by their biological functions. For example, CDHR2 is involved in the surface structure of epithelial cells, which are present in various human organs and related to cancer aetiology [32, 33]. Both ADGRE2 and ADGRD1 are adhesion G protein-coupled receptors involved in cell adhesion and signalling, with ADGRE2 particularly implicated in inflammatory responses of immune system cells [34, 35]. ACY1 is an enzyme that plays a key role in protein breakdown and amino acid salvage, and its deficiency has been linked to metabolic imbalance and developmental delays [36, 37]. Finally, MEGF9 is a transmembrane protein involved in cell adhesion and signalling, with evidence suggesting its role in neurodevelopment [38].

Among all exposures investigated, age yielded the most significant associations with plasma protein levels. The protein most strongly and positively associated with age was EDA2R, a member of the tumour necrosis factor receptor superfamily [39]. EDA2R is involved in cell signalling and tissue development, and its gene expression has been previously linked to ageing in plasma, muscle, and lung tissues [40–42]. Older age was also associated with higher levels of ELN, a protein making up elastic fibres in various human organs, including the skin, heart, and blood vessels [43–46]. Consistent with this finding, we also found a positive association between older age and higher levels of LTBP2, a component of micro-fibrils that interact with ELN [47]. Many age-related proteins were also significantly associated with other exposures. For example, higher levels of CXCL17, a protein involved in homeostasis at mucosal barriers and inflammatory response in respiratory diseases [48, 49], were associated with older age, smoking, and amount of exhaled CO in our study.

Our analyses in the main and mutually adjusted models demonstrated marked sex differences in plasma levels of proteins, including many involved in human reproductive processes, such as FSHB, which regulates follicular growth in females [50, 51], and ACRV1, EDDM3B, and TEX101, which are involved in spermatogenesis in males [52–54]. We replicated the well-known finding of elevated LEP levels in females, a protein released by adipocytes regulating appetite and metabolism, which has been related to sex differences in body fat percentage/distribution [8, 10, 11]. Moreover, we found elevated levels of the XG protein in females, an antigen that defines the Xg blood group [55, 56]. Historically, the Xg blood group has received less research attention, but its strong associations with sex, age, and BMI in our study suggest a need for further investigation into its role in health and disease aetiology. Many of the sex-associated proteins were also independently associated with other exposures, including FSHB, which was also associated with age. Additional analysis revealed this association was primarily driven by female sex, since FSHB regulates the growth of ovarian follicles and elevated FSHB levels are an indicator of menopause [57, 58].

Our analyses also replicated several exposure-protein associations previously reported in Europeans. For example, BMI was associated with 675 proteins in the mutually adjusted model, including known associations of higher BMI with higher levels of LEP (regulating energy balance) [59] and FABP4 (lipid transporter in adipocytes) [60]. Other proteomic associations with clinical measurements included higher SBP and lower REN levels (part of the renin-angiotensin system that regulates blood pressure and fluid balance) [61] and higher RPG and lower LPL levels (involved in lipid metabolism by breaking down triglycerides) [62]. Consistent with previous study findings, we found certain behavioural factors may also be associated with plasma protein levels, such as smoking, associated with higher CXCL17 levels (involved in inflammatory response in respiratory diseases) [48, 49] and alcohol drinking, associated with higher CHI3L1 levels (involved in inflammatory response and liver fibrosis) [63]. However, we found little association of diet and physical activity with plasma levels of proteins. Sample collection factors, including outdoor temperature and fasting time, were also significantly and independently associated with 253 and 83 proteins, respectively. For example, higher outdoor temperature was associated with higher SPINK6 levels (maintaining skin homeostasis and restricting influenza activation) [64, 65], and shorter fasting time was associated with higher GIP levels (stimulating insulin secretion) [66]. These factors may confound exposure-protein associations and should be considered in analyses. Future blood-based studies should also collect relevant information to enhance the robustness and reliability of analyses.

In addition to individual exposures, we identified novel associations between many plasma proteins and two composite measures reflecting general lifestyle and health (i.e., lifestyle index and frailty index). The indices showed opposing associations with some proteins, including IGSF9, IGFBP2, and SHBG. IGSF9 and IGFBP2 are implicated in multiple cancers and are considered potential diagnostic or prognostic markers and treatment targets [67–72]. SHBG, a liver-produced protein that regulates sex hormones [73] is associated with metabolic and reproductive system disorders [74–77]. As expected, many proteins associated with the two indices also showed associations with individual exposures, especially BMI, SBP, and RPG. Sex differences were also observed in proteomic associations with the two indices. For example, lifestyle index showed stronger inverse associations with GGT1 (a liver function marker) [78, 79] and CXCL17 (involved in lung function) [48, 49] in males than females, which may be due to the higher prevalence of alcohol drinking and smoking in males than females (37.2% vs. 2.6% and 63.3% vs. 2.3%, respectively) in CKB and the general Chinese population [80–82].

Apart from leveraging an East Asian population, the main strengths of this study include the large number of proteins assayed, the wide range of exposures considered simultaneously, and the exclusion of participants with prior cardiovascular disease to reduce bias from pre-existing conditions. Consequently, the findings are most directly generalizable to adults free of cardiovascular disease. Additionally, we examined potential sex differences in protein-exposure associations, revealing potential novel findings to inform future research. Nevertheless, the study also had limitations. First, although it is the largest proteomics study in East Asians to date, power was limited for rare binary exposures (e.g., liver disease, cancer, and mental disorder), but was adequate (> 80%; Cohen’s d = 0.3) for binary exposures with prevalence above 25% and high (> 95%; Cohen’s f²=0.023) for continuous exposures. Nonetheless, the study’s findings provide valuable insights in a hypothesis-generating context, although limited power precluded more detailed subgroup analyses beyond sex-specific comparisons. As such, the smaller number of males compared to females might explain fewer significant associations in males observed in our analyses. Second, the cross-sectional study design and lack of repeated measures prevent confirmation of the direction of observed associations. Third, despite extensive adjustment for key covariates to minimise confounding, residual confounding may still persist. Fourth, recall bias in self-reported variables, such as prior diseases and diet, may have affected some associations. However, recall bias is likely to be limited, as three resurveys (~ 5 years apart), each involving ~ 25,000 randomly selected participants from the full CKB cohort, included ~ 15,000 individuals who participated in all three, enabling longitudinal comparisons that suggest good consistency in self-reported data [24, 25]. Therefore, findings should be interpreted with caution, and future studies incorporating longitudinal data and using genetic approaches (e.g., Mendelian Randomisation) are required to clarify causality. Finally, the lack of similar datasets with proteomics data prevented the replication of our findings in independent East Asian cohorts. However, the findings in the current study are also broadly consistent with a study using ~ 7,000 proteins measured by the SomaScan platform in the same sample [83]. A number of top associations were found in both platforms, such as the association between sex and LEP, as well as the association between frailty index and SHBG [83]. The SomaScan study also offers complementary information to the current study, as it covered proteins unique to the SomaScan platform and assessed the influence of other platform-specific factors, such as data normalisation procedures and dilution factors [83]. In addition, over 90% of associations with sex, age, BMI, SBP and diabetes-related exposures were replicated in UK Biobank, supporting generalisability of our findings using the same Olink platform.

Overall, the present study in Chinese adults demonstrated a large number of proteomic associations across a diverse range of exposures, particularly sex, age, adiposity, and frailty indices. We also identified sex differences in proteomic associations with various exposures, mainly reflecting differences in reproductive processes and lifestyle habits between females and males. Future studies from diverse cohorts are still required to replicate our findings, which may guide biomarker discovery when combined with clinical studies. Future analyses using penalisation methods, such as LASSO and elastic net, may be beneficial for building prediction models and reducing the high dimensionality of the exposome and proteomic data [83]. The integration of genetic data with proteomics, such as Mendelian Randomisation and colocalization analyses, can also confirm the causal relevance of these associations and explore biological mechanisms linking exposures to specific proteins.

## Supplementary Information

Below is the link to the electronic supplementary material.

Supplementary Material 1

Supplementary Material 2

## Data Availability

In CKB, non-genetic data (e.g., baseline, resurveys, biomarkers, and disease follow-up) are released periodically to bona fide researchers. Details of the CKB Data Sharing Policy, data release schedules and data request application procedures are available at www.ckbiobank.org. All queries about data access can be made to ckbaccess@ndph.ox.ac.uk

## Abbreviations

ACP5: Tartrate-resistant acid phosphatase type 5
ACRV1: Acrosomal protein SP-10
ACY1: Aminoacylase-1
ADGRD1: Adhesion G-protein coupled receptor D1
ADGRE2: Adhesion G protein-coupled receptor E2
ALPP: ALPP alkaline phosphatase, placental
BMI: Body mass index
C1QA: Complement C1q subcomponent subunit A
CA14: Carbonic anhydrase 14
CDHR2: Cadherin-related family member 2
CES1: Liver carboxylesterase 1
CGA: Glycoprotein hormones alpha chain
CHAD: Chondroadherin
CHI3L1: Chitinase-3-like protein 1
CHRDL2: Chordin-like 2
CKB: China Kadoorie Biobank
CK-BB: Brain-type creatine kinase also known as creatine kinase B-type
COL1A1: Collagen alpha-1(I) chain
CRISP2: Cysteine-rich secretory protein 2
CSF3: Granulocyte colony-stimulating factor receptor
CTRC: Chymotrypsin-C
CTSV: Cathepsin V
CVD: Cardiovascular disease
CXCL17: C-X-C motif chemokine 17
DPP4: Dipeptidyl peptidase-4
DPT: Dermatopontin
EDA2R: Tumor necrosis factor receptor superfamily member 27
EDDM3B: Epididymal secretory protein E3-beta
EGFR: Epidermal growth factor receptor
ELN: Elastin
ERBB3: Receptor tyrosine-protein kinase erbB-3
Exhaled CO: Exhaled carbon monoxide
FABP4: Fatty acid-binding protein, adipocyte
FCGR3B: Low affinity immunoglobulin gamma Fc region receptor III-B
FGF21: Fibroblast growth factor 21
FGF5: Fibroblast growth factor 5
FSHB: Follitropin subunit beta
GDF15: Growth/differentiation factor 15
GGT1: Glutathione hydrolase 1 proenzyme
GHR: Growth hormone receptor
GIP: Gastric inhibitory polypeptide
HAVCR1: Hepatitis A virus cellular receptor 1
HbA1c: Hemoglobin A1c
HBsAg: Hepatitis B virus surface antigen
HS6ST2: Heparan-sulfate 6-O-sulfotransferase 2
HSPB6: Heat shock protein beta-6
IGDCC4: Immunoglobulin superfamily DCC subclass member 4
IGFBP2: Insulin-like growth factor-binding protein 2
IGFBP3: Insulin-like growth factor-binding protein 3
IGSF9: Protein turtle homolog A
IHD: Ischaemic heart disease
IL17D: Interleukin-17D
IL1RN: Interleukin-1 receptor antagonist
INSL3: Insulin-like 3
ITGB5: Integrin beta-5
ITIH4: Inter-alpha-trypsin inhibitor heavy chain H4
KLK3: Prostate-specific antigen
KLK4: Kallikrein-4
LAMP3: Lysosome-associated membrane glycoprotein 3
LEFTY2: Left-right determination factor 2
LEP: Leptin
LPL: Lipoprotein lipase
LRP1: Low-density lipoprotein receptor-related protein 1
LTBP2: Latent-transforming growth factor beta-binding protein 2
MAMDC4: MAM domain-containing 4
MEGF9: Multiple epidermal growth factor-like domains protein 9
MELTF: Melanotransferrin
MSLN: Mesothelin
MSR1: Macrophage scavenger receptor types I and II
NPX: Normalized Protein eXpression
PAEP: Glycodelin
PLAT: Plasminogen activator, tissue type
PON2: Serum paraoxonase/arylesterase 2
pQTLs: Protein quantitative trait locus
PZP: Pregnancy zone protein
QC: Quality control
RELT: Tumor necrosis factor receptor superfamily member 19 L
REN: Renin
RPG: Random plasma glucose
RSPO1: R-spondin-1
SBP: Systolic blood pressure
SCGB3A1: Secretoglobin family 3 A member 1
SD: Standard deviation
SDK2: Protein sidekick-2
SHBG: Sex hormone-binding globulin
SNED1: Sushi, nidogen and EGF-like domain-containing protein 1
SORCS2: VPS10 domain-containing receptor SorCS2
SPESP1: Sperm equatorial segment protein 1
SPINK6: Serine protease inhibitor Kazal-type 6
SPINT3: Kunitz-type protease inhibitor 3
STC2: Stanniocalcin-2
STX7: Syntaxin-7
SUSD5: Sushi domain-containing protein 5
TEX101: Testis-expressed protein 101
TIMP4: Metalloproteinase inhibitor 4
TNFSF11: Tumor necrosis factor ligand superfamily member 11
TNFSF13: Tumor necrosis factor ligand superfamily member 13
VCAN: Versican core protein
VWA1: Von Willebrand factor A domain containing 1
WFDC12: WAP four-disulfide core domain protein 12
XG: Glycoprotein Xg
