# Supplementary material for: Associations of 2923 Olink proteins with demographic, lifestyle, environmental and health characteristics in middle-aged Chinese adults
Source: Eur J Epidemiol. 2025 Oct 10;40(10):1205–20. doi: 10.1007/s10654-025-01311-z (PMC12660334; doi:10.1007/s10654-025-01311-z)
Supplement: Supplementary file 1 — Supplementary Material 1 [file 10654_2025_1311_MOESM1_ESM.docx]

Supplementary Material

**Associations of 2923 Olink proteins with demographic, lifestyle, environmental and health characteristics in Chinese adults**

**Contents**

Members of the China Kadoorie Biobank Collaborative Group 3

eTable 1. List of 2944 protein biomarkers quantified by the Olink Explore I and Olink Explore II assay 4

eTable 2. Baseline characteristics and description of comparison used in analyses 5

eTable 3. Principle component analyses for protein biomarkers and baseline characteristics 7

eTable 4. Baseline characteristics of participants in CKB proteomics’ subcohort and full cohort 8

eTable 5. Effect estimates from the association of baseline characteristics with proteins 9

eTable 6. Number of proteins significantly associated with baseline characteristic after sequential adjustments, in overall analyses 10

eTable 7. Number of proteins significantly associated with baseline characteristic after sequential adjustments, in females 11

eTable 8. Number of proteins significantly associated with baseline characteristic after sequential adjustments, in males 12

eTable 9. Number of proteins significantly associated with only one baseline characteristic, overall and by sex 13

eFigure 1. Study design diagram 14

eFigure 2. Cumulative variance explained by principal components for protein biomarkers and baseline characteristics 15

eFigure 3. Exposure profiles by characteristics type of the top 25 proteins biomarkers with most positive and negative associations, overall and by sex 16

eFigure 4. Age-related protein biomarkers and their associations with other exposures by sex 17

eFigure 5. Comparison of associations of selected baseline characteristics with protein biomarkers between female and male 18

eFigure 6. Associations of selected baseline characteristics with protein biomarkers 19

eFigure 7. Associations of selected baseline characteristics with protein biomarkers by sex 20

eFigure 8. BMI-related protein biomarkers and their associations with other exposures, in overall analyses 21

eFigure 9. BMI-related protein biomarkers and their associations with other exposures, by sex 22

eFigure 10. Post-menopause-related protein biomarkers and their associations with other exposures, in females 23

eFigure 11. Lifestyle index-related protein biomarkers and their associations with other exposures, by sex 24

eFigure 12. Frailty index-related protein biomarkers and their associations with other exposures, by sex 25

eFigure 13. Comparison of protein biomarkers associated with diabetes-related indicators between CKB and UKB 26

eFigure 14. Comparison of associations of key baseline characteristics with protein biomarkers between CKB and UKB, by sex 27

Reference 28

#

# Members of the China Kadoorie Biobank Collaborative Group

**International Steering Committee:** Junshi Chen, Zhengming Chen (PI), Robert Clarke, Rory Collins, Liming Li (PI), Jun Lv, Richard Peto, Robin Walters.

**International Co-ordinating Centre, Oxford:** Daniel Avery, Maxim Barnard, Derrick Bennett, Ruth Boxall, Ka Hung Chan, Yiping Chen, Zhengming Chen, Charlotte Clarke, Jonathan Clarke, Robert Clarke, Huaidong Du, Ahmed Edris Mohamed, Hannah Fry, Simon Gilbert, Pek Kei Im, Andri Iona, Maria Kakkoura, Christiana Kartsonaki, Hubert Lam, Kuang Lin, James Liu, Mohsen Mazidi, Iona Millwood, Sam Morris, Qunhua Nie, Alfred Pozarickij, Maryam Rahmati, Paul Ryder, Dan Schmidt, Becky Stevens, Iain Turnbull, Robin Walters, Baihan Wang, Lin Wang, Neil Wright, Ling Yang, Xiaoming Yang, Pang Yao.

**National Co-ordinating Centre, Beijing:** Xiao Han, Can Hou, Qingmei Xia, Chao Liu, Jun Lv, Pei Pei, Dianjanyi Sun, Canqing Yu, Lang Pan

**10 Regional Co-ordinating Centres:**

Qingdao CDC: Zengchang Pang, Ruqin Gao, Shanpeng Li, Haiping Duan, Shaojie Wang, Yongmei Liu, Ranran Du, Yajing Zang, Liang Cheng, Xiaocao Tian, Hua Zhang, Yaoming Zhai, Feng Ning, Xiaohui Sun, Feifei Li. Licang CDC: Silu Lv, Junzheng Wang, Wei Hou. Heilongjiang Provincial CDC: Wei Sun, Shichun Yan, Xiaoming Cui. Nangang CDC: Chi Wang, Zhenyuan Wu,Yanjie Li, Quan Kang. Hainan Provincial CDC: Huiming Luo, Tingting Ou. Meilan CDC: Xiangyang Zheng, Zhendong Guo, Shukuan Wu, Yilei Li, Huimei Li. Jiangsu Provincial CDC: Ming Wu, Yonglin Zhou, Jinyi Zhou, Ran Tao, Jie Yang, Jian Su. Suzhou CDC: Fang Liu, Jun Zhang, Yihe Hu, Yan Lu, Liangcai Ma, Aiyu Tang, Shuo Zhang, Jianrong Jin, Jingchao Liu. Guangxi Provincial CDC: Mei Lin, Zhenzhen Lu. Liuzhou CDC: Lifang Zhou, Changping Xie, Jian Lan,Tingping Zhu,Yun Liu, Liuping Wei, Liyuan Zhou, Ningyu Chen, Yulu Qin, Sisi Wang. Sichuan Provincial CDC: Xianping Wu, Ningmei Zhang, Xiaofang Chen, Xiaoyu Chang. Pengzhou CDC: Mingqiang Yuan, Xia Wu, Xiaofang Chen, Wei Jiang, Jiaqiu Liu, Qiang Sun. Gansu Provincial CDC: Faqing Chen, Xiaolan Ren, Caixia Dong. Maiji CDC: Hui Zhang, Enke Mao, Xiaoping Wang, Tao Wang, Xi zhang. Henan Provincial CDC: Kai Kang, Shixian Feng, Huizi Tian, Lei Fan. Huixian CDC: XiaoLin Li, Huarong Sun, Pan He, Xukui Zhang. Zhejiang Provincial CDC: Min Yu, Ruying Hu, Hao Wang. Tongxiang CDC: Xiaoyi Zhang, Yuan Cao, Kaixu Xie, Lingli Chen, Dun Shen. Hunan Provincial CDC: Xiaojun Li, Donghui Jin, Li Yin, Huilin Liu, Zhongxi Fu. Liuyang CDC: Xin Xu, Hao Zhang, Jianwei Chen,Yuan Peng, Libo Zhang, Chan Qu.

# eTable 1. List of 2944 protein biomarkers quantified by the Olink Explore I and Olink Explore II assay

Saved into the Excel file as supplementary material.

# eTable 2. Baseline characteristics and description of comparison used in analyses

| **Variables** | **Description** |
| --- | --- |
| **Demographics** |  |
| Age | Participant's age at entry into study |
| Sex | Participant's self-reported sex at entry into the study |
| Urban residents | Is the region participant lives in urban compared to rural? |
| Schooling > 9 years | Does participant have greater than 9 years of education (high school; technical school or college; university) compared to less than 9 years of education (middle school; primary school; no formal education) |
| Occupation | Is the participant employed (employed in specific industry; self-employed) compared to unemployed (retired; house wife/ husband; unemployed; other or not stated)? |
| Income ≥ 20,000 yuan | Is the participant's income ≥ 20,000 yuan annually compared to > 20,000 yuan annually? |
| Ownership index (score out of 6) | Derived by aggregating the following binary variables for each participant: possession of health cover; home ownership; access to private sanitation facilities; access to a motor vehicle; access to a phone; engagement in recent leisure travel |
| **Lifestyle factors** |  |
| Current regular alcohol consumption | Does participant currently consume alcohol regularly (weekly) compared to not (never regular; occasional; monthly; ex-regular; reduced intake) |
| Current regular smoker | Is participant currently a regular smoker (smoker) compared to not (never smoker; occasional smoker; ex-regular)? |
| Food diversity score | Food diversity index with a range of 0-24 calculated from a weighted scoring frequency depending on frequency of consumption of following food groups: dairy; eggs; fish; fresh fruit; fresh vegetables; red meat; wholegrains; poultry; preserved vegetables; rice; soybean; wheat. |
| Rapeseed vs. other plant (excluding lard) | Does participant use rapeseed oil to cook compared to other plant oils (peanut; soybean; other), excluding lard, or not? |
| Physical activity | Number of hours per day engaging in physical activity (MET) |
| **Environmental** |  |
| Outdoor temperature | Mean daily temperature in region (°C) |
| Clean heating fuel | Does participant currently use clean heating fuel (gas; electric; central heating) or not (coal; wood; other)? |
| Clean cooking fuel | Does participant currently use clean cooking fuel (gas or electric) or not (coal; wood; other)? |
| **Health and wellbeing** |  |
| Self-rated health | Does participant have poor self-rated health compared to not poor self-rated health (fair; good; excellent)? |
| Respiratory disease | Has participant self-reported as being doctor diagnosed with any of the following: asthma; COPD; TB; emphysema or bronchitis? |
| Kidney/ liver disease | Has participant self-reported as being doctor diagnosed with any of the following: kidney disease; cirrhosis or hepatitis? |
| HBsAg+ | Has participant tested positive for the presence of hepatitis B surface antigen? (those with 'unclear' result set to missing) |
| Diabetes | Does participant have diabetes or not? This is based on a combination of self-reported doctor diagnosed cases and screen-detected from an RPG test, where an RPG level of ≥ 11.1 mmol/L resulted in a diabetes diagnosis, or if fasting blood glucose was ≥ 7 mmol/L. |
| Cancer | Has participant been diagnosed with cancer by a doctor or not? |
| Life satisfaction | Is participant unsatisfied with their life generally (very unsatisfied; unsatisfied) compared to not being unsatisfied with their life generally (neither satisfied nor unsatisfied; satisfied; very satisfied)? |
| Mental disorder | Has participant been diagnosed with a mental health disorder (major depression; generalised anxiety; other psychiatric disorder)? |
| **Clinical measurements** |  |
| BMI | BMI of participant (kg/ m²), measured at baseline |
| Standing height | Standing height of participant in cm, measured at baseline |
| SBP | Participant's mean systolic blood pressure in mmHg |
| DBP | Participant's mean diastolic blood pressure in mmHg |
| Heart rate | Participant's heart rate in bpm |
| Max CO ppm measurement | Participant's maximum CO measurement |
| FEV1/ FVC ratio | Participant's FEV1/ FVC ratio |
| RBG | Participant's random plasma glucose in mmol/L |
| Fasting time | Participant's fasting time: hours since they last ate |
| **Reproductive factors** |  |
| Age at menarche | Among females, age at which participant had their first period |
| Age at menopause | Among females, age at which menopause began |
| Post-menopausal | Among females, has participant gone through menopause (compared to not menopausal; currently going through menopause) |
| Parity | Among females, has participant ever given birth (live or stillbirth) |
| Age at first live birth | Age at first live birth |
| **Derived indices** |  |
| Healthy lifestyle index | A derived score comprised of baseline variables related to smoking status, alcohol consumption, physical activity, diet and body shape, indicating a healthy lifestyle that were measured by use of self-reported questionnaires, or physical examinations.^1,2^ |
| Frailty index | A proxy measure of accelerated biological aging, comprised of 28 baseline variables indicative of health status deficits that were measured by use of self-reported questionnaires, or physical examinations.^3^ |
| All binary variables in the table are described as yes vs. no (description on left vs. description on right) where 1 = yes; 0 = no. Daily mean weather measurements taken on the same day participant attended assessment centre. BMI: Body mass index; CO: Carbon monoxide; COPD: Chronic obstructive pulmonary disease; DBP: Diastolic blood pressure; FEV1/FVC: Forced expiratory volume in 1 second/ forced vital capacity; MET: Metabolic equivalent task; RPG: Random plasma glucose; SBP: Systolic blood pressure; TB: Tuberculosis | |

# eTable 3. Principle component analyses for protein biomarkers and baseline characteristics

Saved into the Excel file as supplementary material.

# eTable 4. Baseline characteristics of participants in CKB proteomics’ subcohort and full cohort

| **Characteristics^a^** | **Proteomics’ subcohort** | | |  | | **Full cohort^b^** | | | |
| --- | --- | --- | --- | --- | --- | --- | --- | --- | --- |
|  | **Female** (n=1,247) | **Male** (n=759) | **All**  (n=2,006) | |  | | **Female** (n=299,211) | **Male** (n=207,656) | **All**  (n=488,767) |
| **Demographics** |  |  |  | |  | |  |  |  |
| Age, years | 50.7 (10.2) | 50.8 (11.0) | 50.8 (10.5) | |  | | 51.0 (10.4) | 51.1 (10.8) | 51.1 (10.6) |
| Sex | ─ | ─ | ─ | |  | | ─ | ─ | ─ |
| Urban residents | 52.0 | 48.6 | 50.6 | |  | | 43.8 | 42.7 | 43.2 |
| Schooling (>9 years) | 20.4 | 7.6 | 22.9 | |  | | 17.4 | 25.8 | 20.8 |
| Employed | 60.1 | 77.5 | 66.8 | |  | | 63.4 | 80.5 | 70.7 |
| Household income (≥¥20,000) | 43.0 | 47.4 | 44.5 | |  | | 40.9 | 45.6 | 42.8 |
| Ownership index ^c^ | 3.3 (1.3) | 3.4 (1.4) | 3.3 (1.3) | |  | | 3.2 (1.4) | 3.3 (1.4) | 3.3 (1.4) |
| **Lifestyle** |  |  |  | |  | |  |  |  |
| Regular alcohol drinker | 2.6 | 37.2 | 15.3 | |  | | 2.1 | 33.9 | 26.7 |
| Current smoker | 2.3 | 63.3 | 25.3 | |  | | 2.3 | 62.2 | 15.1 |
| Diet |  |  |  | |  | |  |  |  |
| Food diversity score ^d^ | 11.4 (3.3) | 11.3 (3.2) | 11.3 (3.3) | |  | | 10.7 (3.3) | 11.0 (3.2) | 10.8 (3.3) |
| Rapeseed oil | 33.3 | 38.8 | 35.4 | |  | | 38.9 | 39.1 | 38.9 |
| Physical activity, MET-hrs/day | 20.5 (13.2) | 23.2 (16.3) | 21.4 (14.5) | |  | | 20.5 (12.8) | 22.9 (15.3)  0 | 21.5 (13.9) |
| **Environmental** |  |  |  | |  | |  |  |  |
| Outdoor temperature, °C | 16.0 (10.6) | 15.7 (10.9) | 15.9 (10.7) | |  | | 16.2 (10.1) | 15.8 (10.4) | 16.0 (10.2) |
| Clean heating fuel | 45.3 | 44.3 | 45.0 | |  | | 52.9 | 53.8 | 53.2 |
| Clean cooking fuel | 49.9 | 36.0 | 44.8 | |  | | 43.0 | 28.8 | 37.3 |
| **Health and wellbeing** |  |  |  | |  | |  |  |  |
| Self-rated health | 8.5 | 8.2 | 8.3 | |  | | 10.5 | 7.8 | 9.4 |
| Respiratory disease | 8.3 | 8.0 | 8.2 | |  | | 7.4 | 10 | 8.6 |
| Kidney/liver disease | 2.1 | 2.5 | 2.2 | |  | | 2.3 | 3 | 2.6 |
| HBsAg+ | 2.2 | 2.5 | 2.3 | |  | | 2.8 | 3.6 | 3.1 |
| Diabetes | 7.0 | 5.8 | 6.5 | |  | | 5.7 | 5 | 5.4 |
| Cancer | 0.6 | 0.6 | 0.7 | |  | | 0.5 | 0.4 | 0.5 |
| Life satisfaction | 3.7 | 4.9 | 4.0 | | 3.7 | | 3.9 | 4.6 | 4.1 |
| Mental disorder | 1.1 | 1.5 | 1.2 | | 1.1 | | 1.4 | 0.8 | 1.1 |
| **Clinical measurements** |  |  |  | |  | |  |  |  |
| BMI, kg/m² | 24.0 (3.5) | 23.7 (3.3) | 23.9 (3.4) | |  | | 23.7 (3.4) | 23.4 (3.2) | 23.6 (3.4) |
| Standing height, cm | 154.5 (6.1) | 165.8 (6.5) | 158.7 (8.3) | |  | | 154.1 (6.0) | 165.3 (6.5) | 158.7 (8.3) |
| SBP, mmHg | 129.4 (22.2) | 132.6 (19.9) | 130.5 (21.4) | |  | | 129.9 (21.8) | 131.8 (19.8) | 130.6 (21.0) |
| DBP, mmHg | 77.2 (10.6) | 79.7 (11.6) | 78.0 (11.1) | |  | | 76.7 (10.8) | 79.0 (11.3) | 77.6 (11.1) |
| Heart rate, bpm | 79.4 (11.4) | 78.0 (11.9) | 78.8 (11.6) | |  | | 79.7 (11.5) | 77.8 (12.0) | 78.9 (11.8) |
| Exhaled CO, ppm | 5.0 (2.2) | 11.7 (2.5) | 7.5 (2.3) | |  | | 5.1 (2.3) | 11.9 (2.3) | 7.8 (2.3) |
| FEV1/FVC ratio | 85.1 (6.1) | 84.9 (10.1) | 85.0 (8.5) | |  | | 84.8 ( 6.2) | 84.3 (10.4) | 84.6 (8.8) |
| RPG, mmol/L | 6.1 (8.2) | 5.9 (8.8) | 6.0 (8.5) | |  | | 6.1 (8.1) | 5.9 (9.1) | 6.0 (8.5) |
| Fasting time, hours | 5.2 (5.0) | 5.0 (5.0) | 5.1 (5.0) | |  | | 5.1 (5.0) | 4.8 (4.7) | 4.9 (4.9) |
| **Reproductive factors** |  |  |  | |  | |  |  |  |
| Age at menarche, years | 15.4 (2.0) | ─ | 15.4 (2.0) | |  | | 15.4 (2.0) | ─ | 15.4 (2.0) |
| Age at menopause, years | 39.2 (4.3) | ─ | 39.2 (4.3) | |  | | 44.9 (4.4) | ─ | 39.2 (4.3) |
| Post-menopausal | 54.7 | ─ | 54.7 | |  | | 55.9 | ─ | 54.7 |
| Parity | 99.8 | ─ | 99.8 | |  | | 99.7 | ─ | 99.8 |
| Age at first live birth, years | 23.9 (3.3) | ─ | 23.9 (3.3) | |  | | 23.4 (3.2) | ─ | 23.9 (3.3) |
| **Lifestyle index** ^e^ | 3.1 (0.8) | 2.2 (1.0) | 2.8 (1.0) | |  | | 3.1 (0.8) | 2.2 (1.0) | 2.7 (1.0) |
| **Frailty index** ^f^ | 0.1 (0.06) | 0.1 (0.06) | 0.1 (0.06) | |  | | 0.1 (0.06) | 0.1 (0.06) | 0.1 (0.06) |
| ^a^ Baseline characteristics adjusted for age (10-year age groups) and study area (10 regions).  ^b^ Excluding 23,955 participants with prior CVD or taking statins.  ^c^ 6-point index of qualitative measures of living standards  ^d^ 24-point index of frequency of intake in 12 food groups  ^e^ 5-point index of low-risk lifestyle characteristics  ^f^ 28-point index of accumulation of health deficits and physical activity  Abbreviations: BMI: Body mass index; CO: carbon-monoxide; DBP: Diastolic blood pressure; FEV1/FVC: Forced Expiratory Volume in 1 second / Forced Vital Capacity; HBsAg+: Hepatitis B virus surface antigen seropositive; MET: metabolic equivalent task; RPG: random plasma glucose | | | | | | | | | |

# eTable 5. Effect estimates from the association of baseline characteristics with proteins

Saved into the Excel file as supplementary material.

# eTable 6. Number of proteins significantly associated with baseline characteristic after sequential adjustments, in overall analyses

| **Characteristics** | **Adjustments** | | | | | |
| --- | --- | --- | --- | --- | --- | --- |
|  | **Basic^a^** | **+ fasting time^b^** | **+ outdoor temperature^c^** | **+ BMI^d^** | **+ SBP^e^** | **+ mutual^f^** |
| **Demographics** |  |  |  |  |  |  |
| Age, years | 1123 | 1120 | 1154 | 1158 | 986 | 766 |
| Sex | 799 | 799 | 827 | 842 | 838 | 467 |
| Urban residents | 360 | 359 | 359 | 336 | 311 | 107 |
| Schooling | 3 | 3 | 3 | 2 | 2 | 0 |
| Employed | 3 | 2 | 3 | 4 | 0 | 2 |
| Household income | 3 | 9 | 9 | 2 | 5 | 1 |
| Ownership index ^g^ | 13 | 13 | 15 | 2 | 2 | 0 |
| **Lifestyle** |  |  |  |  |  |  |
| Regular alcohol drinker | 80 | 80 | 75 | 81 | 76 | 67 |
| Current smoker | 54 | 52 | 53 | 61 | 64 | 23 |
| Diet |  |  |  |  |  |  |
| Food diversity score ^h^ | 2 | 1 | 1 | 1 | 1 | 0 |
| Rapeseed oil | 21 | 22 | 22 | 20 | 20 | 12 |
| Physical activity | 2 | 2 | 3 | 4 | 4 | 1 |
| **Environmental** |  |  |  |  |  |  |
| Outdoor temperature | 277 | 277 | 292 | 298 | 247 | 253 |
| Clean heating fuel | 1 | 1 | 1 | 0 | 0 | 0 |
| Clean cooking fuel | 0 | 0 | 0 | 0 | 0 | 0 |
| **Health and wellbeing** |  |  |  |  |  |  |
| Self-rated health | 1 | 1 | 3 | 4 | 4 | 2 |
| Respiratory disease | 0 | 0 | 0 | 0 | 0 | 0 |
| Kidney/liver disease | 0 | 0 | 0 | 0 | 0 | 0 |
| HBsAg+ | 275 | 275 | 282 | 285 | 285 | 295 |
| Diabetes | 340 | 331 | 340 | 249 | 214 | 212 |
| Cancer | 1 | 1 | 1 | 2 | 2 | 2 |
| Life satisfaction | 0 | 0 | 0 | 0 | 0 | 0 |
| Mental disorder | 0 | 0 | 0 | 0 | 0 | 0 |
| **Clinical measurements** |  |  |  |  |  |  |
| BMI | 850 | 860 | 869 | 869 | 700 | 675 |
| Standing height | 17 | 19 | 18 | 17 | 16 | 15 |
| SBP | 565 | 561 | 479 | 206 | 206 | 157 |
| DBP | 431 | 420 | 380 | 144 | 144 | 129 |
| Heart rate | 229 | 248 | 234 | 206 | 206 | 160 |
| Exhaled CO | 26 | 26 | 29 | 29 | 30 | 7 |
| FEV1/FVC ratio | 2 | 1 | 1 | 0 | 0 | 0 |
| RPG | 355 | 371 | 387 | 315 | 268 | 54 |
| Fasting time | 108 | 108 | 79 | 89 | 87 | 83 |
| **Reproductive factors** |  |  |  |  |  |  |
| Age at menarche | 1 | 1 | 1 | 0 | 0 | 0 |
| Age at menopause | 2 | 2 | 2 | 1 | 1 | 1 |
| Post-menopausal | 184 | 182 | 181 | 185 | 185 | 170 |
| Parity | 4 | 4 | 4 | 4 | 4 | 4 |
| Age at first live birth | 0 | 0 | 0 | 0 | 0 | 0 |
| **Lifestyle index** ^i^ | 353 | 335 | 342 | 342 | 271 | 188 |
| **Frailty index** ^j^ | 597 | 594 | 597 | 597 | 597 | 573 |

^a^ Analyses are adjusted for age, age^2^, sex, study area, and plate ID, where appropriate. Bonferroni (PCA) corrected p-value < 0.05

^b^ fasting time and fasting time^2^

^c^ Analyses are additionally adjusted for outdoor temperature and outdoor temperature^2^, where appropriate.

^d^ Analyses are additionally adjusted for BMI, where appropriate.

^e^ Analyses are additionally adjusted for SBP, where appropriate.

^f^ Analyses are additionally adjusted for education, employment, income, ownership index, alcohol, smoking, food diversity score, physical activity, self-rated health, diabetes, life satisfaction, mental disorder, exhaled CO, where appropriate.

^g^ 6-point index of qualitative measures of living standards

^h^ 24-point index of frequency of intake in 12 food groups

^i^ 5-point index of low-risk lifestyle characteristics

^j^ 28-point index of accumulation of health deficits and physical activity

Abbreviations: BMI: Body mass index; CO: carbon-monoxide; DBP: Diastolic blood pressure; HBsAg+: Hepatitis B virus surface antigen seropositive; RPG: random plasma glucose

# eTable 7. Number of proteins significantly associated with baseline characteristic after sequential adjustments, in females

| **Characteristics** | **Adjustments** | | | | | |
| --- | --- | --- | --- | --- | --- | --- |
|  | **Basic^a^** | **+ fasting time^b^** | **+ outdoor temperature^c^** | **+ BMI^d^** | **+ SBP^e^** | **+ mutual^f^** |
| **Demographics** |  |  |  |  |  |  |
| Age, years | 1162 | 1146 | 1133 | 1103 | 918 | 738 |
| Urban residents | 236 | 231 | 224 | 231 | 200 | 56 |
| Schooling | 3 | 4 | 4 | 2 | 2 | 1 |
| Employed | 1 | 1 | 1 | 0 | 0 | 0 |
| Household income | 1 | 1 | 1 | 1 | 1 | 1 |
| Ownership index ^g^ | 0 | 0 | 0 | 0 | 0 | 0 |
| **Lifestyle** |  |  |  |  |  |  |
| Regular alcohol drinker | 0 | 0 | 0 | 0 | 0 | 0 |
| Current smoker | 4 | 5 | 5 | 5 | 5 | 5 |
| Diet |  |  |  |  |  |  |
| Food diversity score ^h^ | 0 | 0 | 0 | 0 | 0 | 0 |
| Rapeseed oil | 6 | 7 | 6 | 7 | 7 | 5 |
| Physical activity | 1 | 0 | 1 | 2 | 1 | 0 |
| **Environmental** |  |  |  |  |  |  |
| Outdoor temperature | 178 | 174 | 174 | 176 | 117 | 127 |
| Clean heating fuel | 0 | 0 | 0 | 0 | 0 | 0 |
| Clean cooking fuel | 0 | 0 | 1 | 0 | 0 | 0 |
| **Health and wellbeing** |  |  |  |  |  |  |
| Self-rated health | 178 | 0 | 0 | 1 | 1 | 1 |
| Respiratory disease | 0 | 0 | 0 | 0 | 0 | 0 |
| Kidney/liver disease | 0 | 0 | 0 | 0 | 0 | 0 |
| HBsAg+ | 186 | 191 | 198 | 202 | 207 | 219 |
| Diabetes | 220 | 216 | 217 | 174 | 138 | 138 |
| Cancer | 2 | 2 | 2 | 2 | 3 | 2 |
| Life satisfaction | 0 | 0 | 1 | 0 | 1 | 1 |
| Mental disorder | 0 | 0 | 0 | 0 | 0 | 0 |
| **Clinical measurements** |  |  |  |  |  |  |
| BMI | 588 | 595 | 576 | 576 | 436 | 418 |
| Standing height | 6 | 6 | 6 | 6 | 6 | 3 |
| SBP | 371 | 364 | 295 | 118 | 118 | 69 |
| DBP | 191 | 182 | 178 | 62 | 62 | 55 |
| Heart rate | 57 | 57 | 57 | 53 | 53 | 31 |
| Exhaled CO | 0 | 0 | 0 | 0 | 0 | 0 |
| FEV1/FVC ratio | 0 | 0 | 0 | 0 | 0 | 0 |
| RPG | 232 | 259 | 254 | 179 | 149 | 24 |
| Fasting time | 55 | 55 | 56 | 58 | 58 | 57 |
| **Reproductive factors** |  |  |  |  |  |  |
| Age at menarche | 1 | 1 | 1 | 0 | 0 | 0 |
| Age at menopause | 2 | 2 | 2 | 1 | 1 | 1 |
| Post-menopausal | 184 | 182 | 181 | 185 | 185 | 170 |
| Parity | 4 | 4 | 4 | 4 | 4 | 4 |
| Age at first live birth | 0 | 0 | 0 | 0 | 0 | 0 |
| **Lifestyle index** ^i^ | 109 | 103 | 99 | 99 | 77 | 46 |
| **Frailty index** ^j^ | 286 | 281 | 279 | 279 | 279 | 262 |

^a^ Analyses are adjusted for age, age^2^, study area, and plate ID, where appropriate. Bonferroni (PCA) corrected p-value < 0.05

^b^ fasting time and fasting time^2^

^c^ Analyses are additionally adjusted for outdoor temperature and outdoor temperature^2^, where appropriate.

^d^ Analyses are additionally adjusted for BMI, where appropriate.

^e^ Analyses are additionally adjusted for SBP, where appropriate.

^f^ Analyses are additionally adjusted for education, employment, income, ownership index, alcohol, smoking, food diversity score, physical activity, self-rated health, diabetes, life satisfaction, mental disorder, exhaled CO, where appropriate.

^g^ 6-point index of qualitative measures of living standards

^h^ 24-point index of frequency of intake in 12 food groups

^i^ 5-point index of low-risk lifestyle characteristics

^j^ 28-point index of accumulation of health deficits and physical activity

Abbreviations: BMI: Body mass index; CO: carbon-monoxide; DBP: Diastolic blood pressure; HBsAg+: Hepatitis B virus surface antigen seropositive; RPG: random plasma glucose

# eTable 8. Number of proteins significantly associated with baseline characteristic after sequential adjustments, in males

| **Characteristics** | **Adjustments** | | | | | |
| --- | --- | --- | --- | --- | --- | --- |
|  | **Basic^a^** | **+ fasting time^b^** | **+ outdoor temperature^c^** | **+ BMI^d^** | **+ SBP^e^** | **+ mutual^f^** |
| **Demographics** |  |  |  |  |  |  |
| Age, years | 625 | 623 | 612 | 610 | 561 | 351 |
| Urban residents | 198 | 206 | 185 | 101 | 100 | 40 |
| Schooling | 0 | 0 | 0 | 0 | 0 | 0 |
| Employed | 0 | 0 | 0 | 0 | 0 | 0 |
| Household income | 9 | 11 | 11 | 8 | 9 | 0 |
| Ownership index ^g^ | 26 | 28 | 29 | 13 | 13 | 0 |
| **Lifestyle** |  |  |  |  |  |  |
| Regular alcohol drinker | 67 | 68 | 53 | 64 | 56 | 50 |
| Current smoker | 49 | 48 | 47 | 46 | 51 | 7 |
| Diet |  |  |  |  |  |  |
| Food diversity score ^h^ | 0 | 0 | 0 | 0 | 0 | 0 |
| Rapeseed oil | 0 | 0 | 0 | 0 | 0 | 0 |
| Physical activity | 0 | 0 | 0 | 0 | 0 | 0 |
| **Environmental** |  |  |  |  |  |  |
| Outdoor temperature | 61 | 57 | 57 | 59 | 46 | 43 |
| Clean heating fuel | 0 | 0 | 0 | 0 | 0 | 0 |
| Clean cooking fuel | 0 | 0 | 0 | 0 | 0 | 0 |
| **Health and wellbeing** |  |  |  |  |  |  |
| Self-rated health | 0 | 0 | 0 | 0 | 0 | 0 |
| Respiratory disease | 1 | 1 | 2 | 2 | 2 | 1 |
| Kidney/liver disease | 2 | 2 | 2 | 2 | 2 | 1 |
| HBsAg+ | 46 | 46 | 45 | 47 | 49 | 52 |
| Diabetes | 39 | 42 | 39 | 37 | 36 | 37 |
| Cancer | 1 | 1 | 1 | 1 | 1 | 1 |
| Life satisfaction | 0 | 0 | 0 | 0 | 0 | 0 |
| Mental disorder | 0 | 0 | 0 | 0 | 0 | 0 |
| **Clinical measurements** |  |  |  |  |  |  |
| BMI | 353 | 355 | 353 | 353 | 265 | 264 |
| Standing height | 0 | 0 | 0 | 0 | 0 | 0 |
| SBP | 92 | 93 | 80 | 17 | 17 | 10 |
| DBP | 115 | 119 | 108 | 45 | 45 | 28 |
| Heart rate | 47 | 47 | 41 | 39 | 39 | 29 |
| Exhaled CO | 37 | 38 | 35 | 35 | 35 | 6 |
| FEV1/FVC ratio | 1 | 1 | 1 | 0 | 0 | 0 |
| RPG | 80 | 84 | 84 | 87 | 86 | 26 |
| Fasting time | 26 | 26 | 26 | 27 | 27 | 27 |
| **Lifestyle index** ^i^ | 132 | 134 | 126 | 126 | 96 | 69 |
| **Frailty index** ^j^ | 98 | 102 | 102 | 102 | 102 | 103 |

^a^ Analyses are adjusted for age, age^2^, study area, and plate ID, where appropriate. Bonferroni (PCA) corrected p-value < 0.05

^b^ fasting time and fasting time^2^

^c^ Analyses are additionally adjusted for outdoor temperature and outdoor temperature^2^, where appropriate.

^d^ Analyses are additionally adjusted for BMI, where appropriate.

^e^ Analyses are additionally adjusted for SBP, where appropriate.

^f^ Analyses are additionally adjusted for education, employment, income, ownership index, alcohol, smoking, food diversity score, physical activity, self-rated health, diabetes, life satisfaction, mental disorder, exhaled CO, where appropriate.

^g^ 6-point index of qualitative measures of living standards

^h^ 24-point index of frequency of intake in 12 food groups

^i^ 5-point index of low-risk lifestyle characteristics

^j^ 28-point index of accumulation of health deficits and physical activity

Abbreviations: BMI: Body mass index; CO: carbon-monoxide; DBP: Diastolic blood pressure; HBsAg+: Hepatitis B virus surface antigen seropositive; RPG: random plasma glucose

# eTable 9. Number of proteins significantly associated with only one baseline characteristic, overall and by sex

| **Characteristics** | **Female** | **Male** | **All** |
| --- | --- | --- | --- |
| **Demographics** |  |  |  |
| Age | 394 | 352 | 168 |
| Sex | 0 | 0 | 77 |
| Urban residents | 39 | 57 | 31 |
| Ownership index | 0 | 3 | 0 |
| **Lifestyle** |  |  |  |
| Regular alcohol drinker | 0 | 14 | 3 |
| Current smoker | 1 | 8 | 0 |
| **Environmental** |  |  |  |
| Outdoor temperature | 97 | 36 | 97 |
| **Health and wellbeing** |  |  |  |
| Physical health |  |  |  |
| Respiratory disease | 0 | 1 | 0 |
| Kidney/liver disease | 0 | 1 | 0 |
| HBsAg+ | 17 | 8 | 12 |
| Diabetes | 1 | 0 | 1 |
| Cancer | 1 | 1 | 1 |
| **Clinical measurements** |  |  |  |
| BMI | 59 | 117 | 37 |
| SBP | 10 | 0 | 3 |
| DBP | 0 | 5 | 0 |
| Heart rate | 0 | 6 | 1 |
| Exhaled CO | 0 | 1 | 0 |
| RPG | 8 | 21 | 2 |
| Fasting time | 2 | 9 | 1 |
| **Reproductive factors** |  |  |  |
| Age at menopause | 1 | ─ | 0 |
| Post-menopausal | 2 | ─ | 1 |
| Parity | 1 | ─ | 1 |
| **Lifestyle index** | 0 | 13 | 0 |
| **Frailty index** | 4 | 5 | 3 |
| Analyses are adjusted for age, age^2^, sex, study area, fasting time, fasting time^2^, outdoor temperature, outdoor temperature^2^ and plate ID, where appropriate. Bonferroni (PCA) corrected p-value < 0.05  Abbreviations: BMI: Body mass index; CO: carbon-monoxide; DBP: Diastolic blood pressure; HBsAg+: Hepatitis B virus surface antigen seropositive; RPG: random plasma glucose | | | |

# eFigure 1. Study design diagram

*19 individuals include both in MI and fatal non-MI IHD cases

**29 individuals included in both IHD cases and subcohort

#
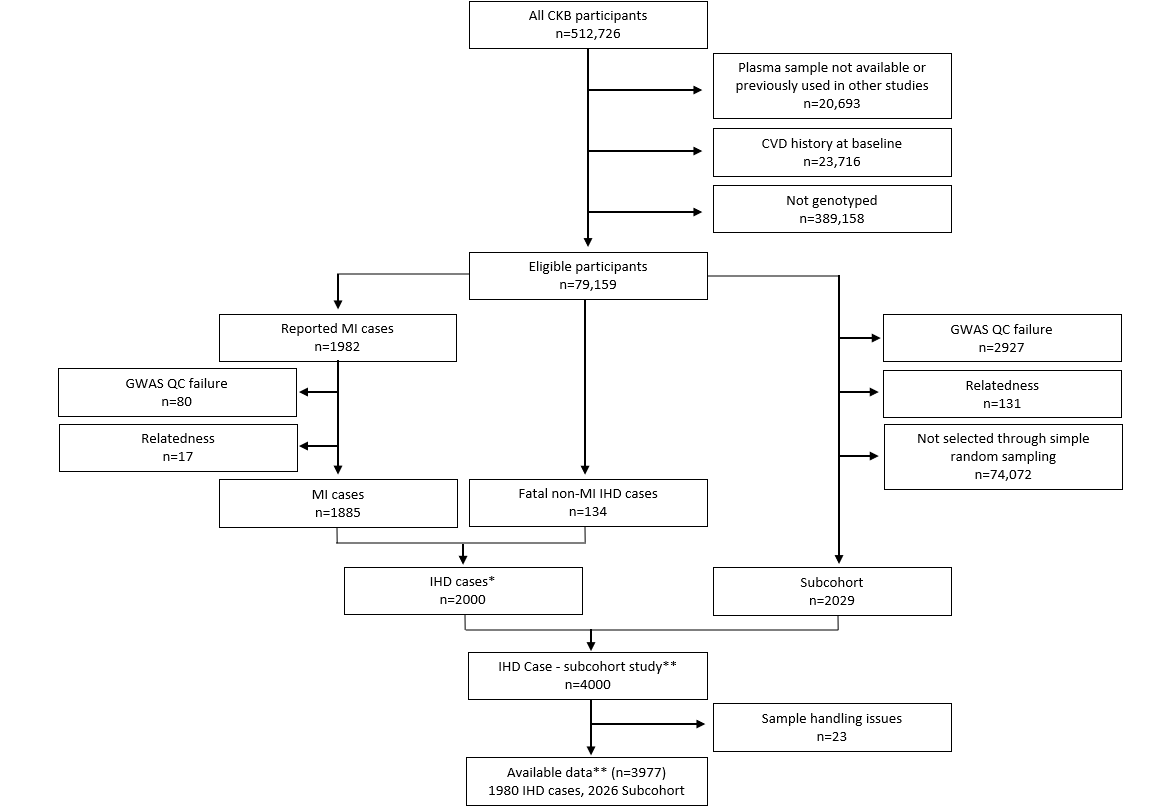


# eFigure 2. Cumulative variance explained by principal components for protein biomarkers and baseline characteristics

The cumulative variance explained (%) is plotted against the number of principal components (red curve). The individual variance explained by each component is represented by bar chart. The blue dashed line indicates the point at which 90% of the variance is reached. Abbreviation: PC: principal component

.
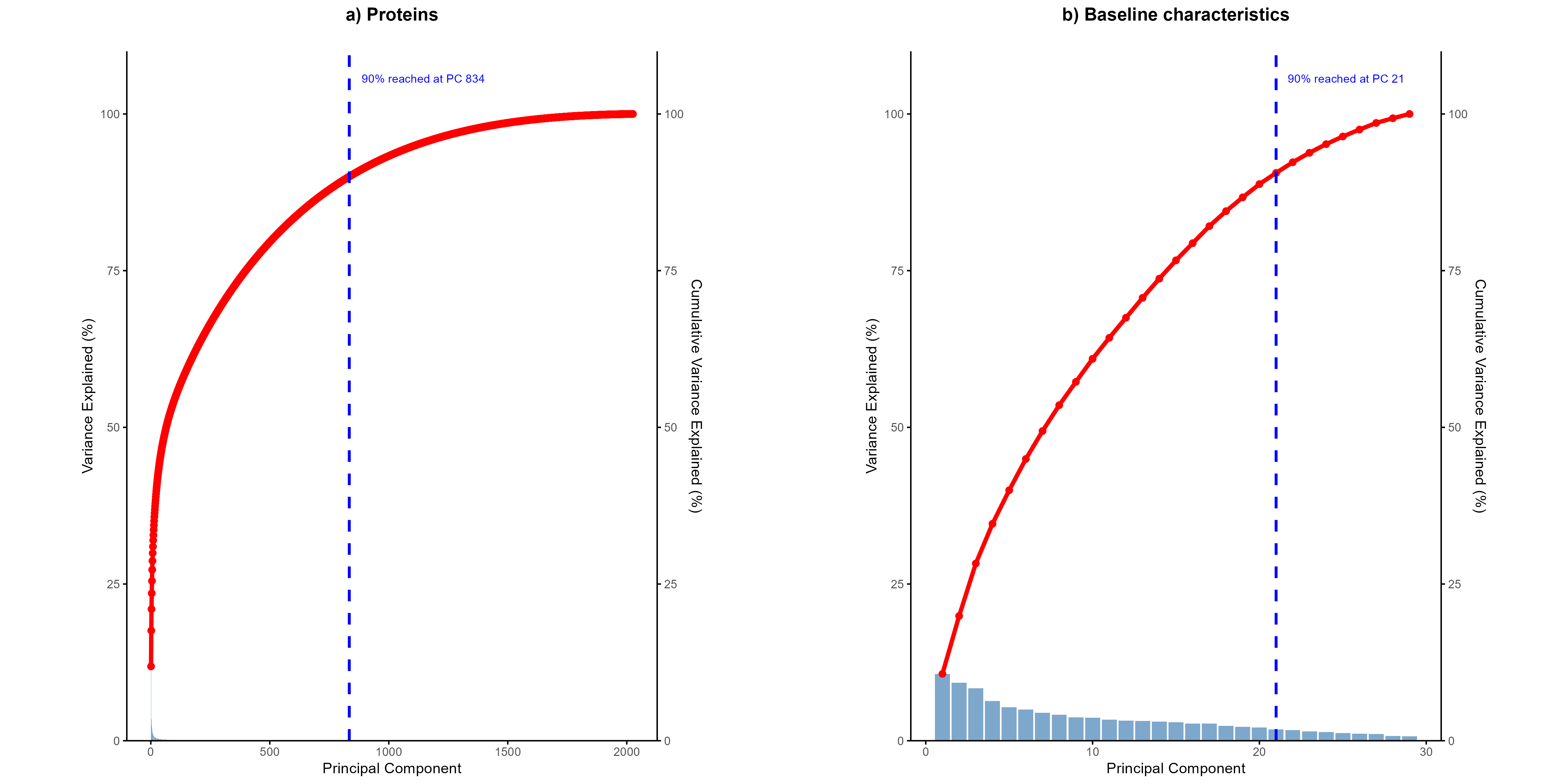


# eFigure 3. Exposure profiles by characteristics type of the top 25 proteins biomarkers with most positive and negative associations, overall and by sex

The bar plots show the number of baseline characteristics positively and negatively associated with the most frequently associated protein biomarkers after Bonferroni corrected p-value. The analyses are presented separately for females, males, and the overall. The 25 protein biomarkers with the most negative associations and the 25 protein biomarkers with the most positive associations are presented. The x-axis represents the protein biomarkers, while the y-axis indicates the number of baseline characteristics associated with each protein (left: negatively- and right: positively-associated). Bars are color-coded to represent different baseline characteristic groups. Analyses are adjusted for age, age^2^, sex, study area, fasting time, fasting time^2^, outdoor temperature outdoor temperature^2^, and plate ID where appropriate.


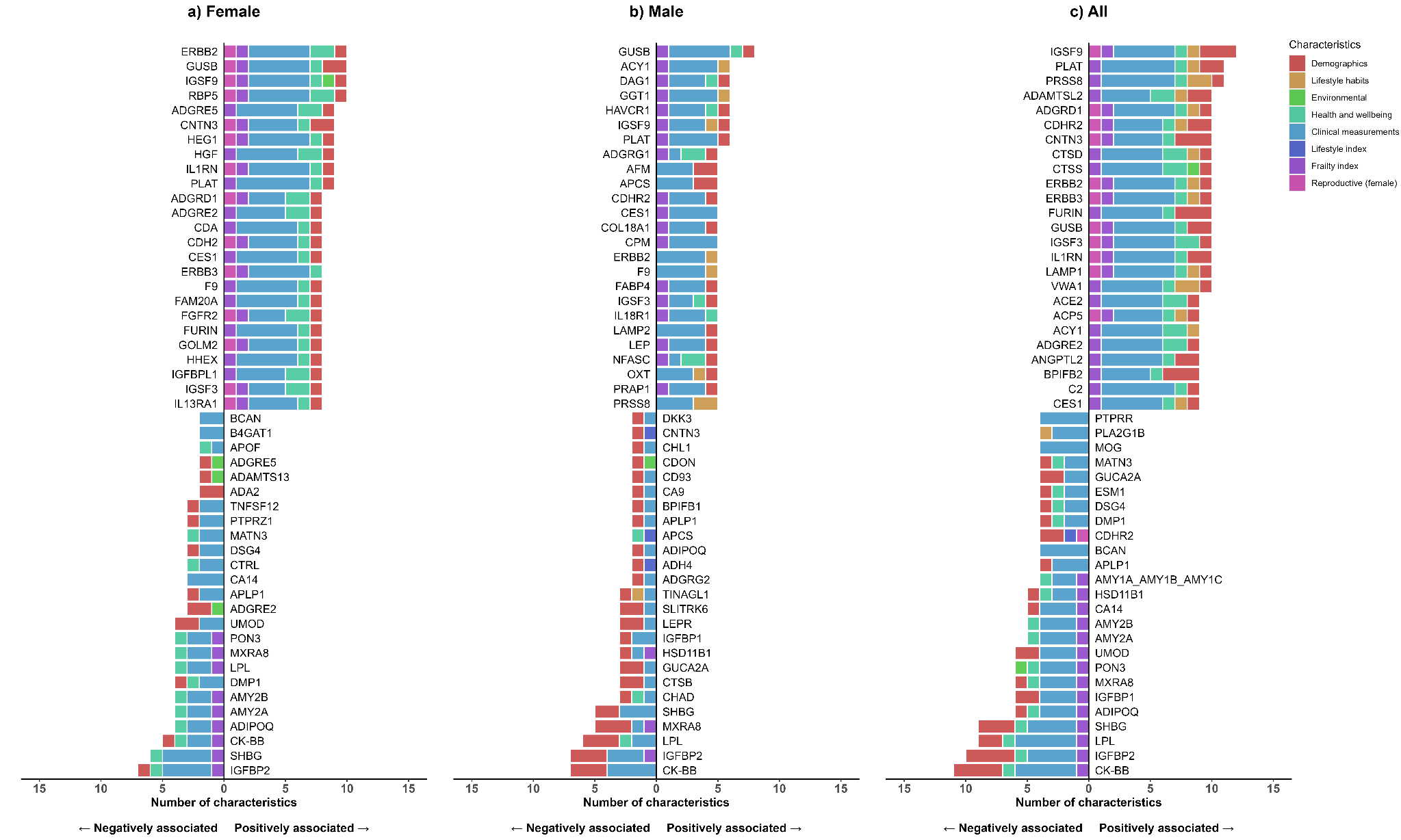


# eFigure 4. Age-related protein biomarkers and their associations with other exposures by sex

Figures a) and c) represent the associations of age in female and male, respectively, with protein biomarkers. The x-axis represents the effect size of the association between age and the protein biomarkers, while the y-axis indicates the –log10 p-value. Red dots denote positive Bonferroni corrected associations, blue dots denote negative Bonferroni corrected associations, and grey dots denote non-significant associations. Figures b) and d) illustrate the top age-associated protein biomarkers, respectively, and their associations with other exposures. The width of the ribbons is inversely proportional to the p-value, indicating the strength of the association (smaller p-values correspond to wider ribbons). The colors of the ribbons represent different baseline characteristic groups. The top protein biomarkers that are not associated with other exposures are not presented in the figure. Analyses are adjusted for age, age^2^, sex, study area, fasting time, fasting time^2^, outdoor temperature, outdoor temperature^2^ and plate ID, where appropriate.

Abbreviations: BMI: Body mass index; CO: carbon-monoxide; DBP: Diastolic blood pressure; HBSAg+: Hepatitis B virus surface antigen seropositive; RPG: random plasma glucose

~~
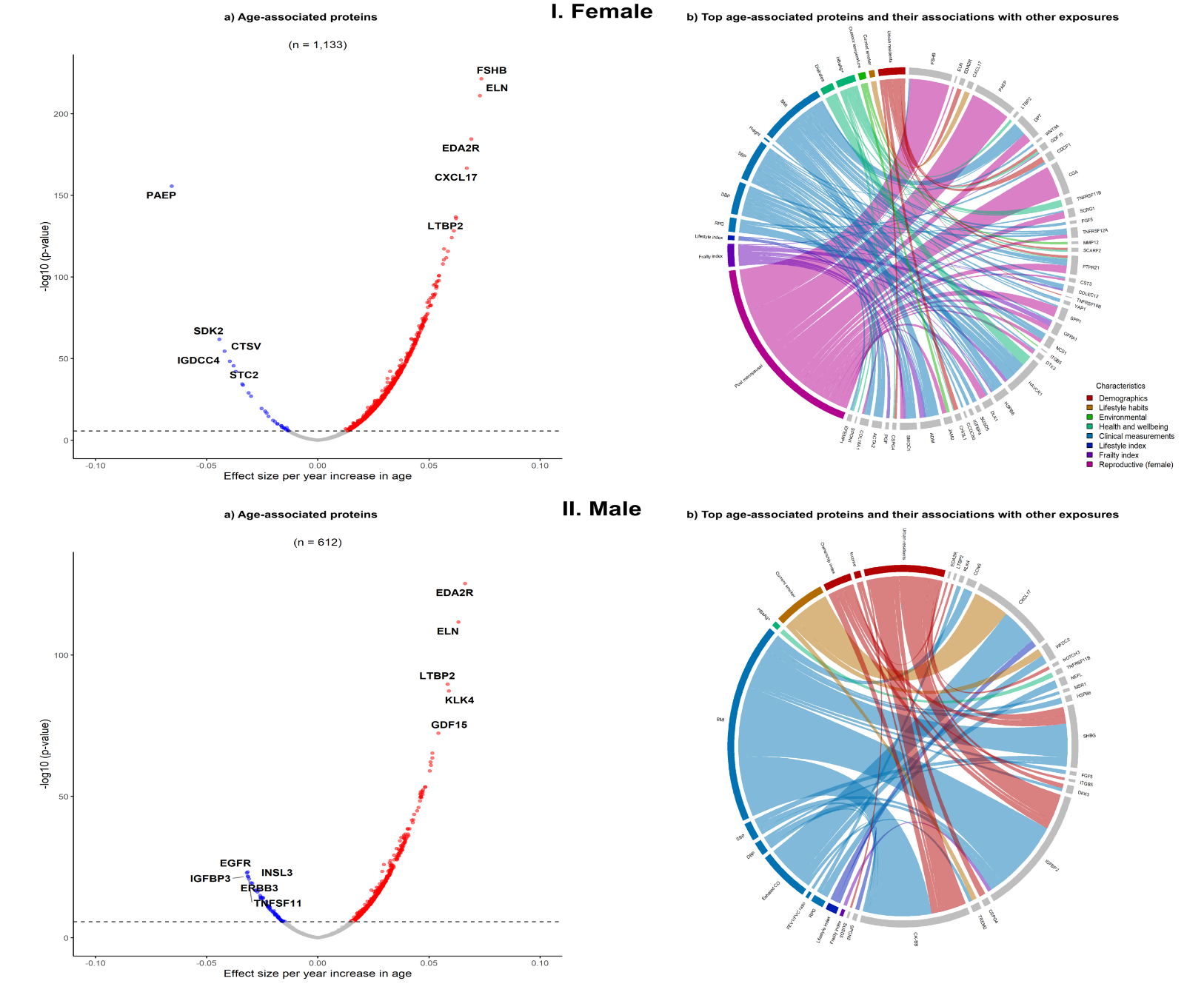
~~

# eFigure 5. Comparison of associations of selected baseline characteristics with protein biomarkers between female and male

Abbreviations: BMI: Body mass index; HBV: Hepatitis B virus; SBP: systolic blood pressure; RPG: Random plasma glucose


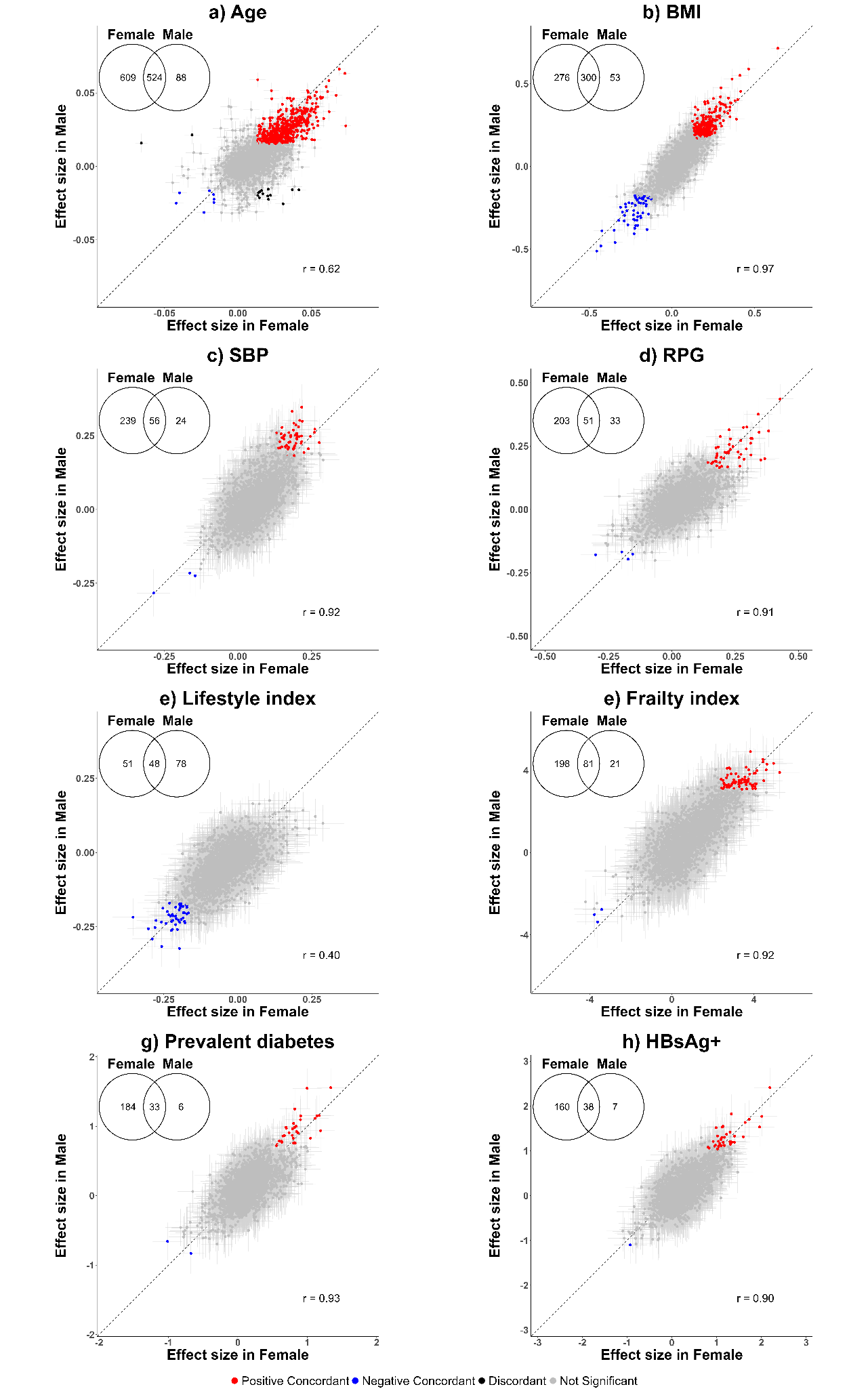


# eFigure 6. Associations of selected baseline characteristics with protein biomarkers

Abbreviations: HBsAg+: Hepatitis B virus surface antigen seropositive; SBP: systolic blood pressure; RPG: Random plasma glucose


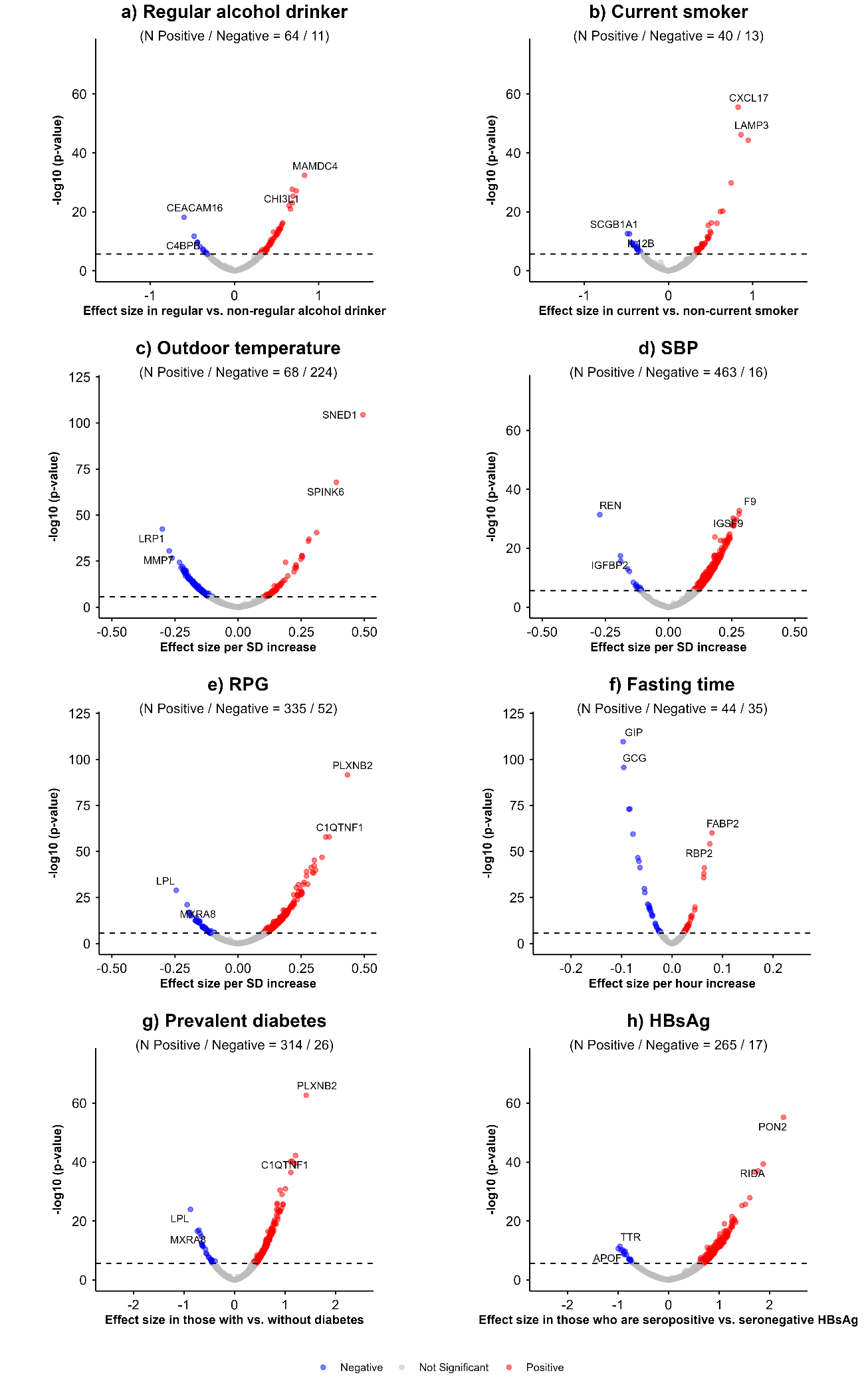


# eFigure 7. Associations of selected baseline characteristics with protein biomarkers by sex

Abbreviations: HBsAg+: Hepatitis B virus surface antigen seropositive; SBP: systolic blood pressure; RPG: Random plasma glucose


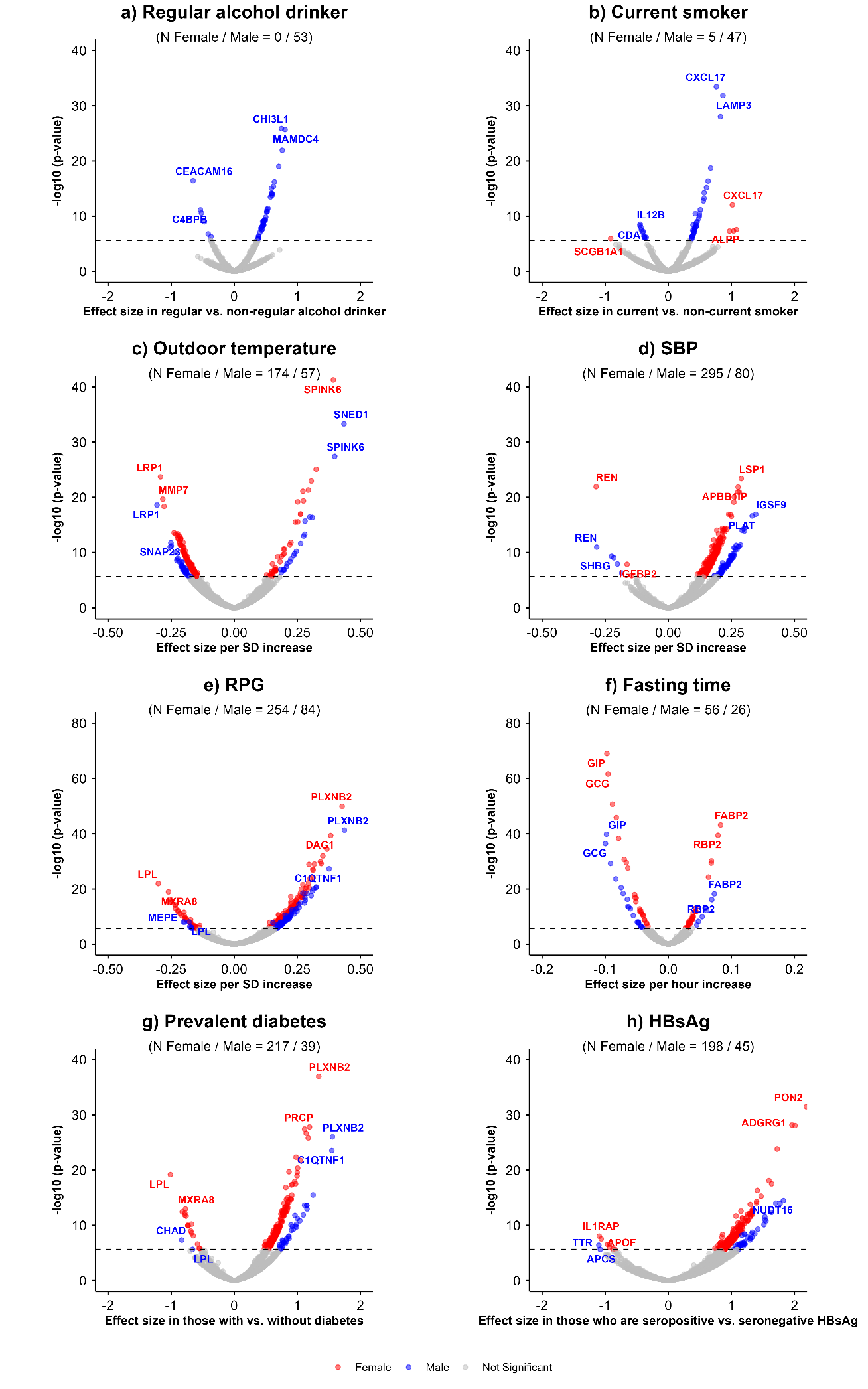


# eFigure 8. BMI-related protein biomarkers and their associations with other exposures, in overall analyses

Legend conventions as eFigure 3.

~~
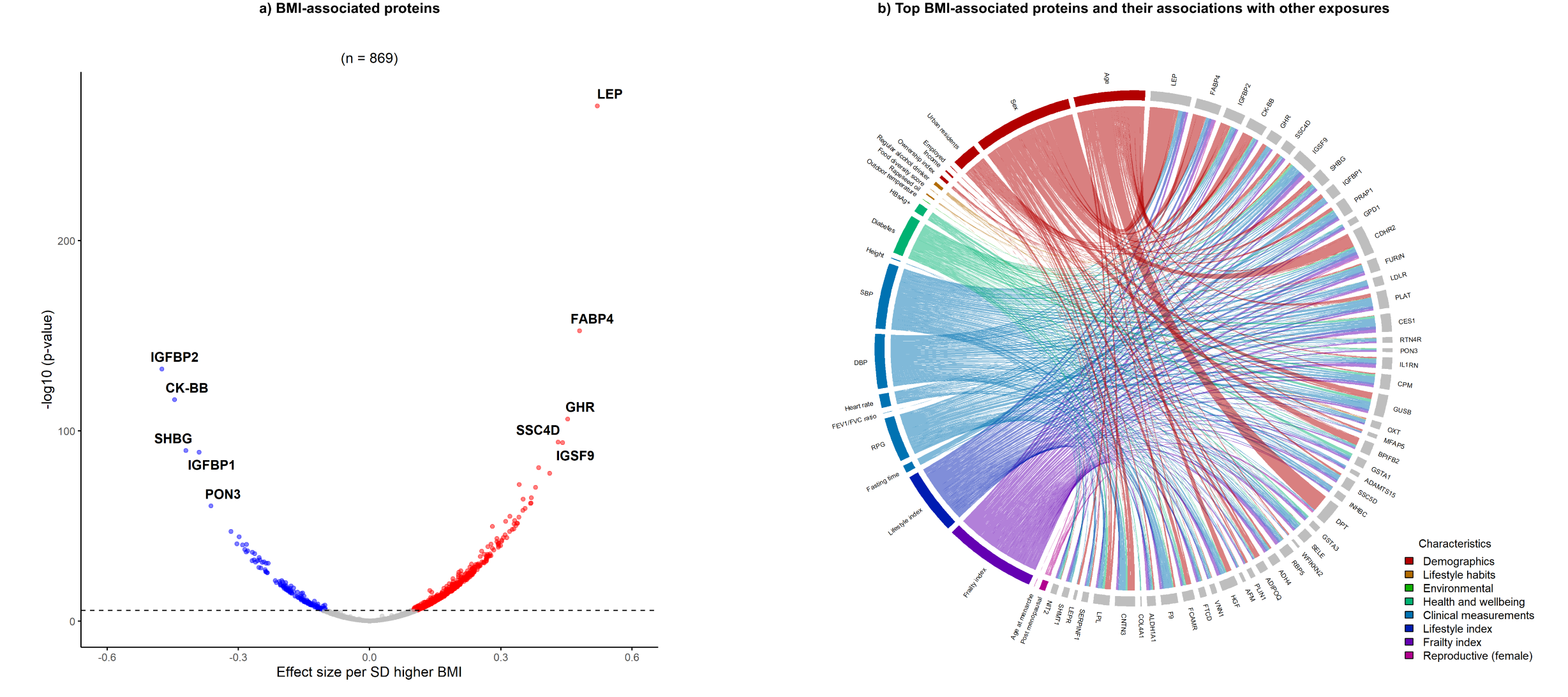
~~

# eFigure 9. BMI-related protein biomarkers and their associations with other exposures, by sex

Legend conventions as eFigure 3.


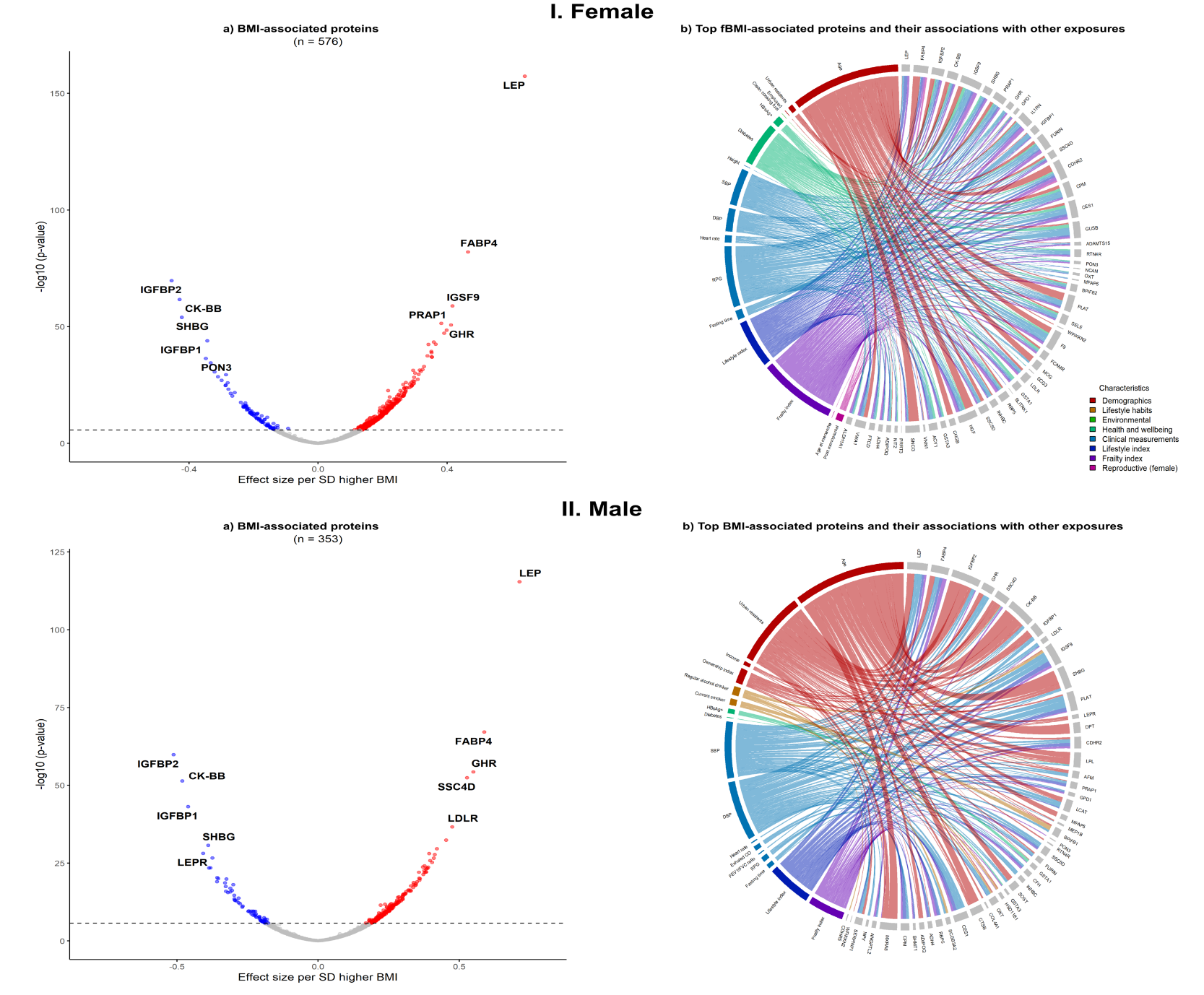


# eFigure 10. Post-menopause-related protein biomarkers and their associations with other exposures, in females

Legend conventions as eFigure 3.

~~
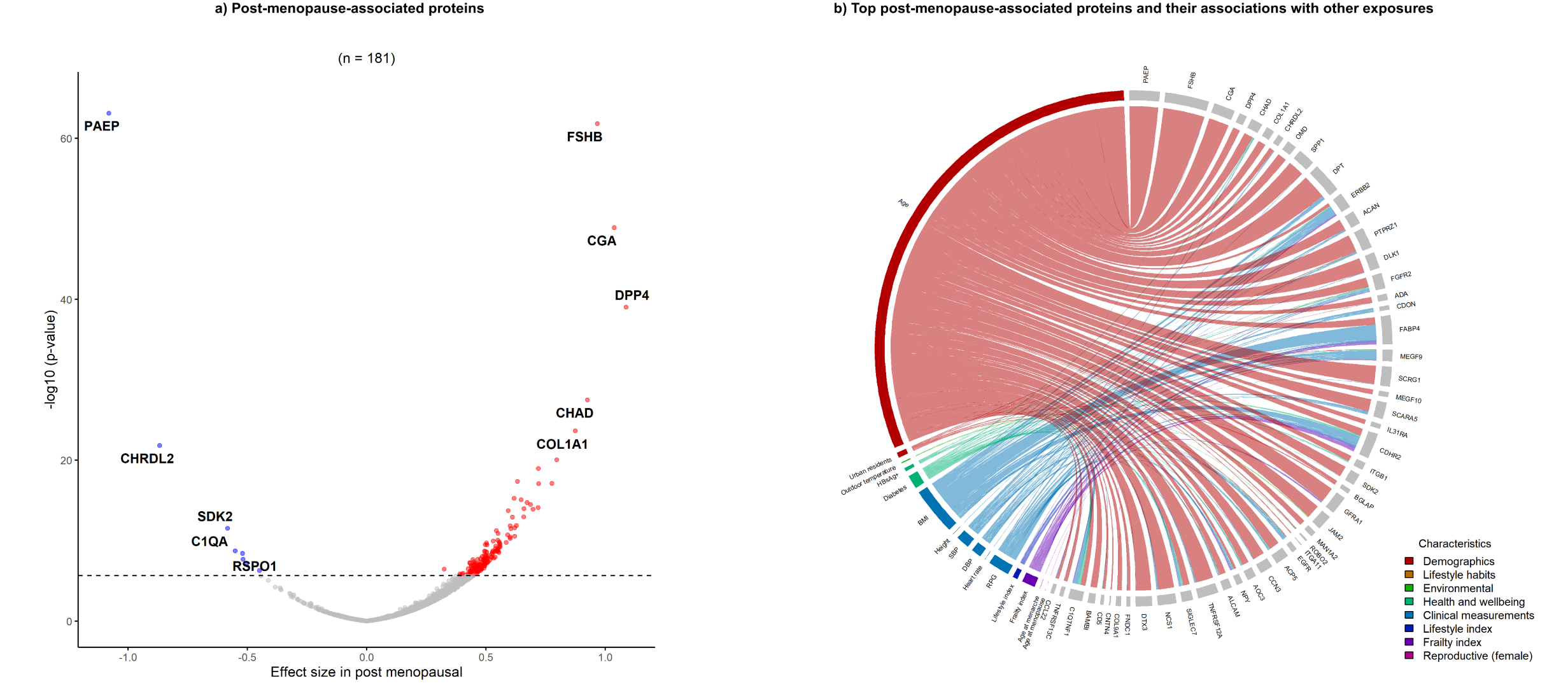
~~

# eFigure 11. Lifestyle index-related protein biomarkers and their associations with other exposures, by sex

Legend conventions as eFigure 3.


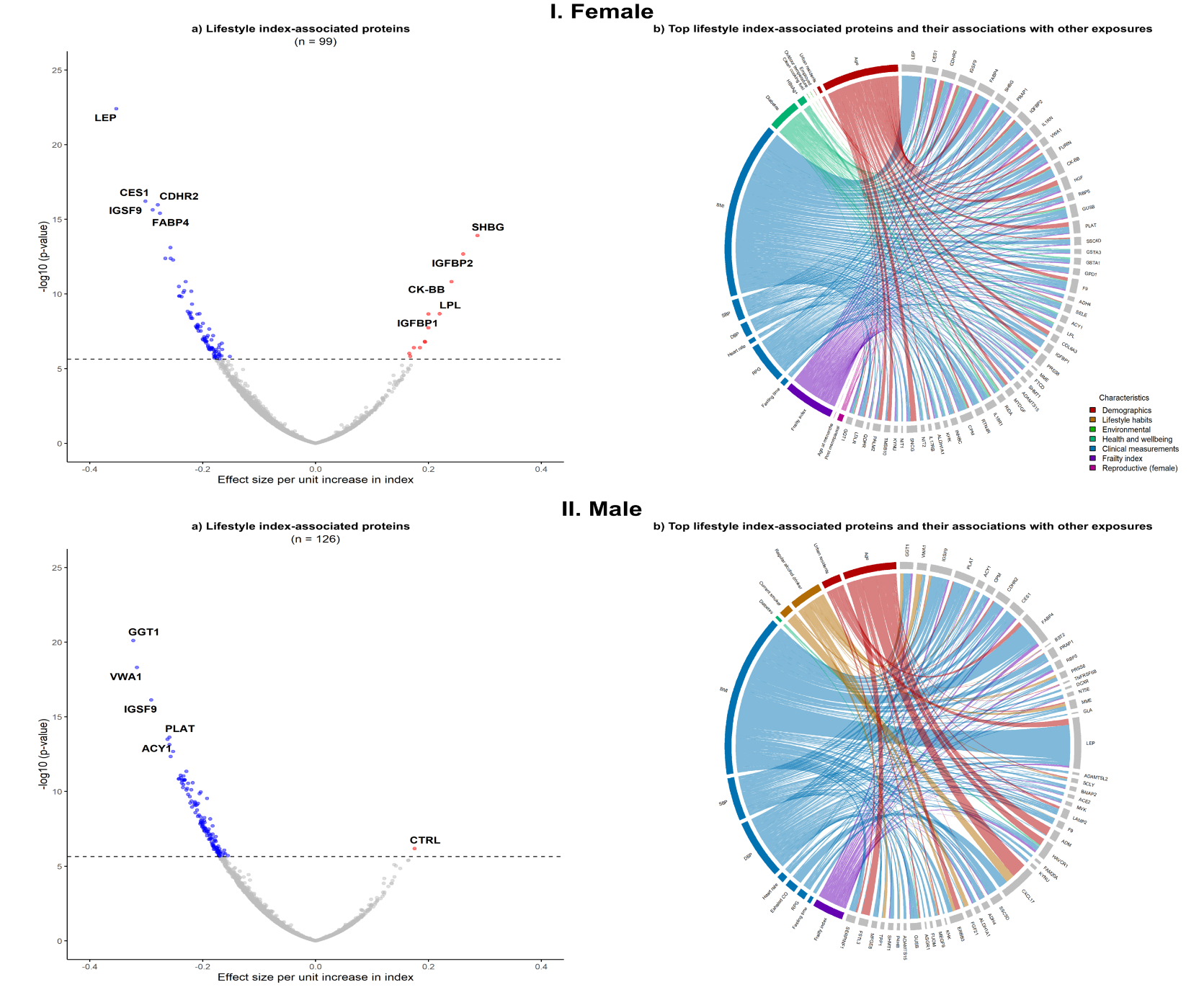


# eFigure 12. Frailty index-related protein biomarkers and their associations with other exposures, by sex

Legend conventions as eFigure 3.


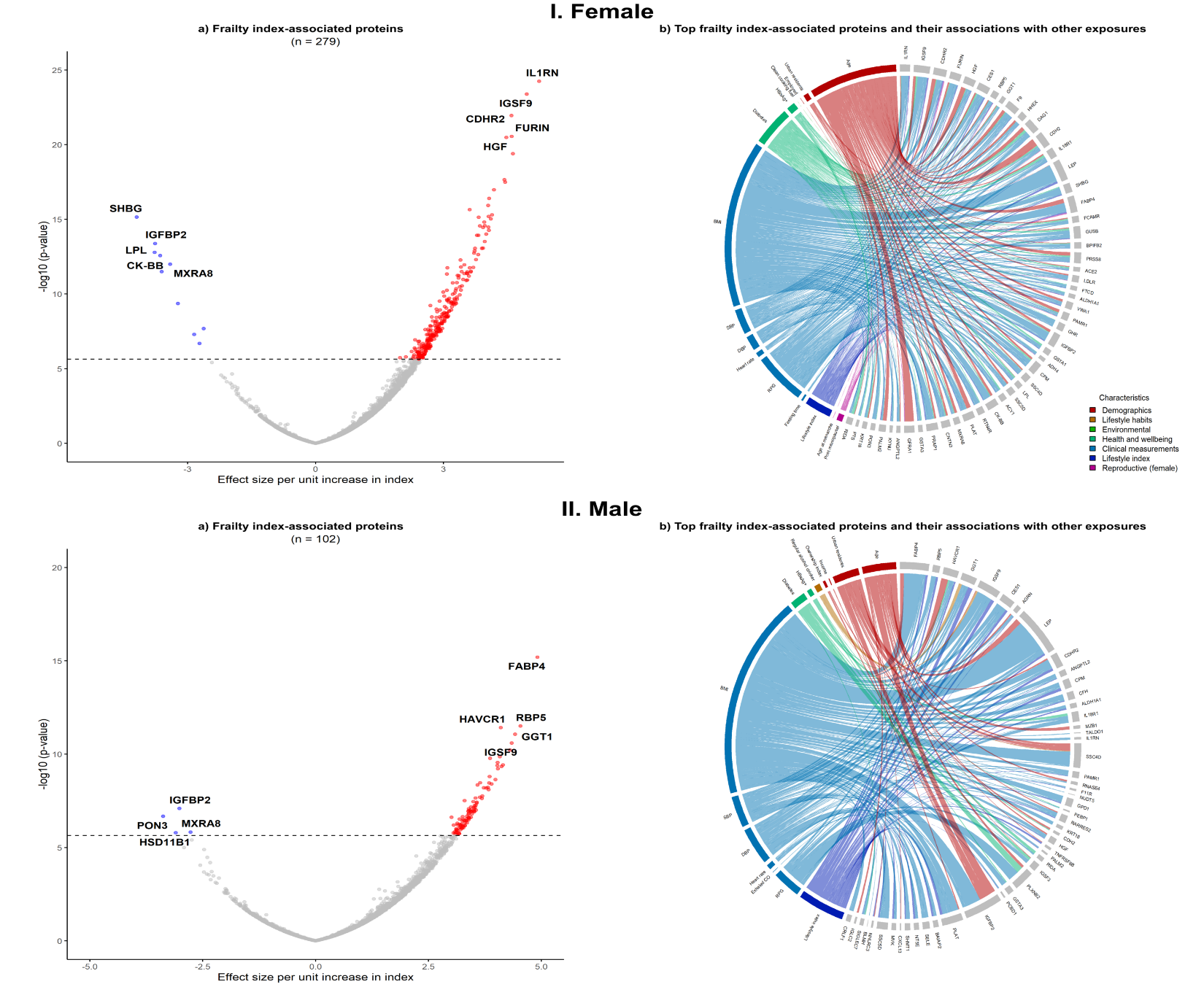


# eFigure 13. Comparison of protein biomarkers associated with diabetes-related indicators between CKB and UKB

In CKB, analyses were adjusted for age, age^2^, sex, study area, fasting time, fasting time^2^, outdoor temperature, outdoor temperature^2^ and plate ID, where appropriate. In UKB, analyses were adjusted for age, age^2^, sex, assessment centre, fasting time, fasting time^2^, season, and plate ID, where appropriate.

Abbreviations: CKB: China Kadoorie Biobank; HbA1c: Hemoglobin A1c; UKB: UK Biobank; RPG: Random plasma glucose


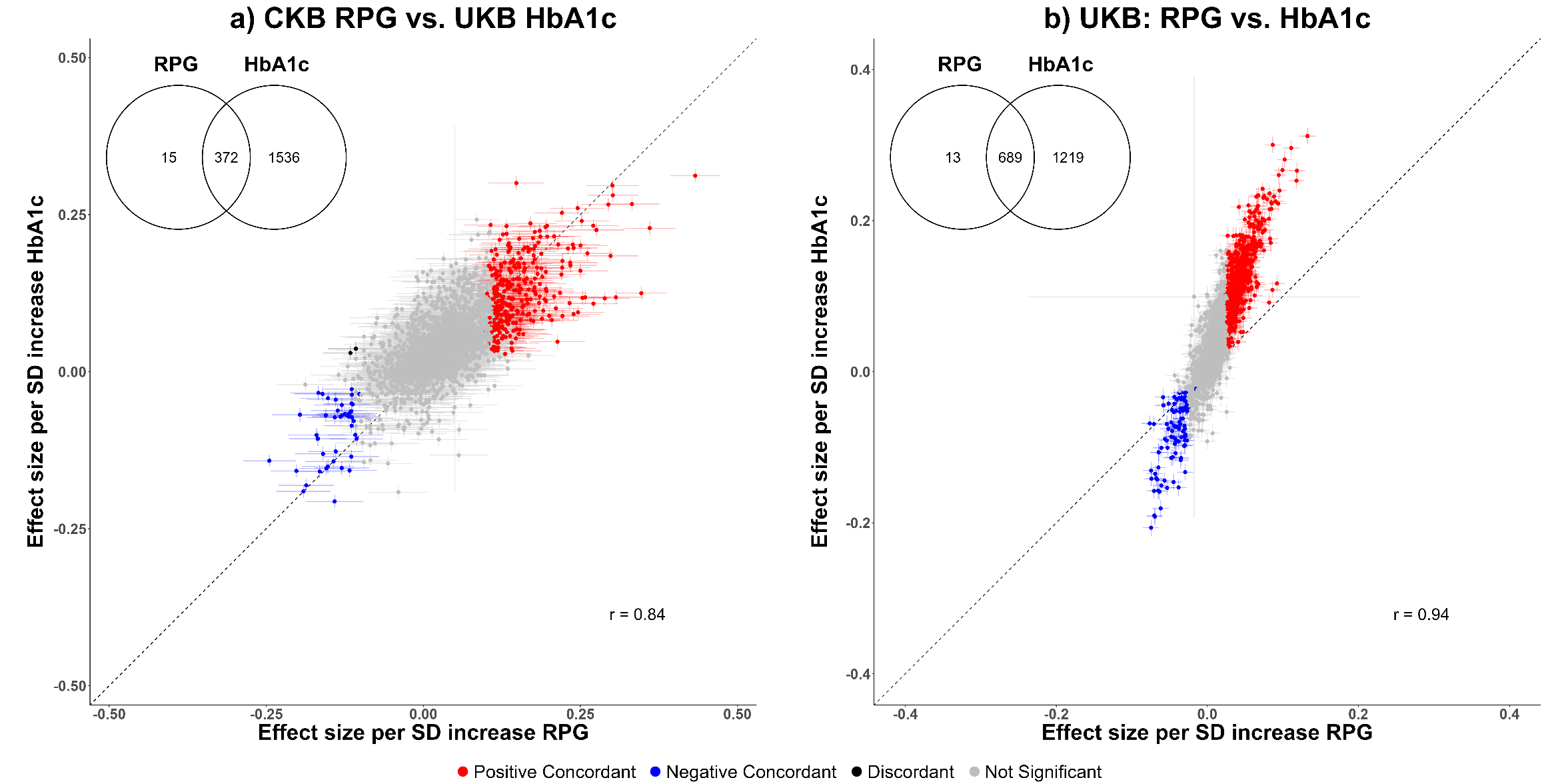


# eFigure 14. Comparison of associations of key baseline characteristics with protein biomarkers between CKB and UKB, by sex

In CKB, analyses were adjusted for age, age^2^, study area, fasting time, fasting time^2^, outdoor temperature, outdoor temperature^2^ and plate ID, where appropriate. In UKB, analyses were adjusted for age, age^2^, assessment centre, fasting time, fasting time^2^, season, and plate ID, where appropriate.

Abbreviations: BMI: Body mass index; CKB: China Kadoorie Biobank; SBP: systolic blood pressure; UKB: UK Biobank; RPG: Random plasma glucose


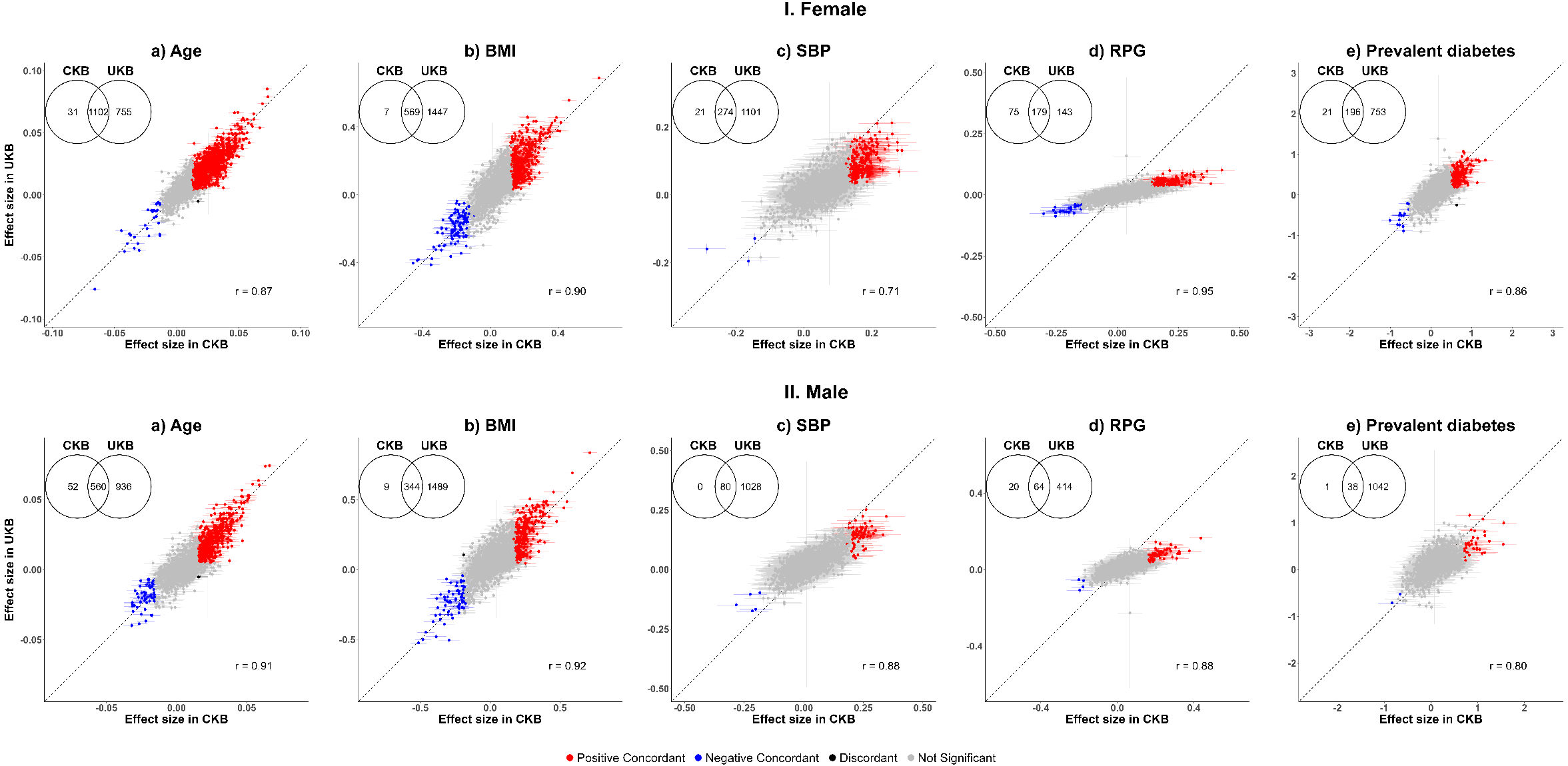


# Reference

1. Lv J, Yu C, Guo Y, Bian Z, Yang L, Chen Y, et al. Adherence to a healthy lifestyle and the risk of type 2 diabetes in Chinese adults. Int J Epidemiol. 2017;46(5):1410-20.

2. Sun Q, Yu D, Fan J, Yu C, Guo Y, Pei P, et al. Healthy lifestyle and life expectancy at age 30 years in the Chinese population: an observational study. Lancet Public Health. 2022;7(12):e994-e1004.

3. Fan J, Yu C, Guo Y, Bian Z, Sun Z, Yang L, et al. Frailty index and all-cause and cause-specific mortality in Chinese adults: a prospective cohort study. Lancet Public Health. 2020;5(12):e650-e60.
